# Digital exclusion and cognitive impairment in older people: findings from five longitudinal studies

**DOI:** 10.1186/s12877-024-05026-w

**Published:** 2024-05-07

**Authors:** Yuge Wang, Zhigang Wu, Lanzhi Duan, Sijia Liu, Ruzhao Chen, Tao Sun, Jiang Wang, Jianghua Zhou, Hongxia Wang, Pan Huang

**Affiliations:** 1 College of Nursing, Wenzhou Medical University, Wenzhou, China; 2 Department of Epidemiology and Health Statistics, Xiangya School of Public Health, Central South University, Changsha, Hunan China; 3 Center for Clinical Medicine Research of Jinggangshan University, Affiliated Hospital of Jinggangshan University, Ji’an, China; 4 Department of Medicine, JingGangshan University, Ji’an, China; 5 Online Collaborative Research Center for Evidence-Based Medicine Ministry of Education, JingGangshan Univesity, Ji’an, China; 6 Department of Cardiology, The First Affiliated Hospital of Wenzhou Medical University, Wenzhou, China; 7 Department of Nursing, The First Affiliated Hospital of Wenzhou Medical University, Wenzhou, China

**Keywords:** Digital exclusion, Cognitive impairment, Older people

## Abstract

**Objectives:**

Older people are more likely to have digital exclusion, which is associated with poor health. This study investigated the relationship between digital exclusion and cognitive impairment in older adults from 23 countries across five longitudinal surveys.

**Design and measurements:**

Digital exclusion is defined as self-reported non-use of the Internet. We assessed cognitive impairment on three dimensions: orientation, memory, and executive function. We used generalized estimation equations fitting binary logistic regression with exchangeable correlations to study the relationship between digital exclusion and cognitive impairment, and apply the minimum sufficiently adjusted set of causally directed acyclic graphs as the adjusted variable.

**Setting and participants:**

We pooled a nationally representative sample of older adults from five longitudinal studies, including the China Health and Retirement Longitudinal study (CHARLS), the English Longitudinal Study of Ageing (ELSA), the Health and Retirement Study (HRS), the Mexican Health and Ageing Study (MHAS) and the Survey of Health, Ageing and Retirement in European (SHARE).

**Results:**

We included 62,413 participants from five longitudinal studies. Digital exclusion varied by country, ranging from 21.69% (SHARE) in Denmark to 97.15% (CHARLS) in China. In the original model, digital exclusion was significantly associated with cognitive impairment in all five studies. In the adjusted model, these associations remained statistically significant: CHARLS (Odds ratio [OR] = 2.81, 95% confidence interval [CI] 1.84–4.28, ELSA (1.92 [1.70–2.18]), HRS(2.48[2.28–2.71), MHAS (1.92 [1.74–2.12]), and SHARE (2.60 [2.34–2.88]).

**Conclusion:**

Our research shows that a significant proportion of older people suffer from digital exclusion, especially in China. Digital exclusion was positively correlated with cognitive impairment. These findings suggest that digital inclusion could be an important strategy to improve cognitive function and reduce the risk of cognitive impairment in older adults.

**Supplementary Information:**

The online version contains supplementary material available at 10.1186/s12877-024-05026-w.

## Introduction

Cognitive decline was once considered the most common and feared side of the aging process [1], which can cause serious health problems and financial burdens to families and health care systems [2–4]. Digital exclusion, which is the lack of access to or ability to use information and communication technologies, is also common among the elderly [5]. In the context of the rapid development of the Internet, the act of seeking health information and receiving medical services online has become increasingly common [6, 7], but many older adults do not have access to the internet or the skills to use it [8], this can lead to negative health outcomes, including cognitive impairment. Therefore, this study is dedicated to investigate the relationship between digital exclusion and cognitive impairment.

Lu et al. [9] explored the relationship between digital exclusion and functional dependence in five cohorts, suggesting that older people excluded from the Internet are more likely to develop functional dependence, regardless of whether they live in high-income or lower-middle-income countries. In addition, Liu et al. investigated the impact of digital rejection on cognitive impairment in Chinese adults and found a positive correlation between digital rejection and cognitive impairment in Chinese adults [10]. However, the study’s participants were limited to the Chinese population, the results cannot be extrapolated to other countries, and the CHARLS database includes four waves of data, but the authors only used data from the fourth wave, ignoring the first three waves. There remains relatively little research on digital exclusion and cognitive dysfunction in the older population over 60 years of age.

We found that digital exclusion was significantly associated with cognitive impairment in older adults. This suggests that digital inclusion could be an important strategy to improve cognitive health in older adults. Consequently, we aimed to investigate the relationship between digital exclusion and cognitive impairment using multiple databases (CHARLS, ELSA, HRS, MHAS, SHARE), namely, older populations of different ethnic groups.

## Method

### Study design and population

This study was conducted in accordance with the Strengthening the Reporting of Observational Studies in Epidemiology (STROBE) guidelines, which aim to improve the quality of reporting of observational research. We adhered to the STROBE checklist throughout the preparation of this manuscript to ensure transparent and complete reporting of our study methods, results, and conclusions (Supplementary materials).

Data were obtained from five longitudinal studies: the China Health and Retirement Longitudinal Study (CHARLS) [11], English Longitudinal Study of Ageing (ELSA) [12], Health and Retirement Study (HRS) [13], Mexican Health and Aging Study (MHAS) [14], and the Survey of Health, Ageing and Retirement in Europe (SHARE) [15]. In this study, we used partial data from five longitudinal studies: w1-w4 (2011–2018) for CHARLS, w7-w9 (2014–2018) for ELSA,w10-w13 (2010–2016) for HRS, w4-w5 (2015–2018) for MHAS, w5-w7 (2013–2017) for SHARE. More details of these five longitudinal studies can be found in the Supplementary materials. The five surveys used in this study were designed to obtain comparable results.

**Fig. 1:**
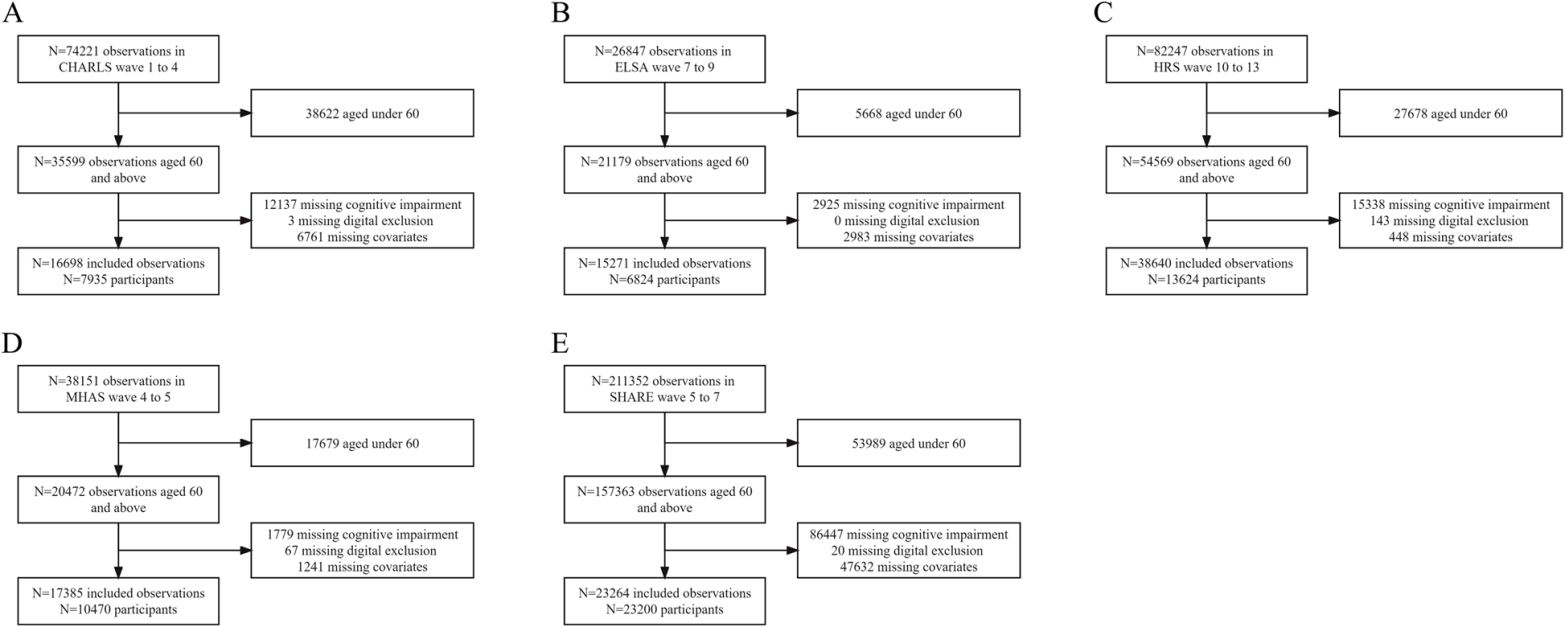
The flowchart of the study population screening

We excluded participants younger than 60 years of age and those with missing data regarding digital exclusion, cognitive impairment, or covariates. Finally, 7935 participants from CHARLS, 6824 from ELSA, 13,624 from HRS, 10,470 from MHAS, and 23,560 from SHARE were included in the analysis. The detailed screening process is illustrated in Fig. 1.

### Digital exclusion

Data on digital exclusion were collected using self-administered questionnaires. In CHARLS, digital exclusion was assessed using the question “Have you used the Internet in the past month?”. For ELSA, the participants were asked how often they use the Internet, with responses ranging from 1 = ‘‘every day, or almost every day’’ to 6 = ‘‘never’’ For HRS, digital exclusion was assessed by the question ‘‘Do you regularly use the Internet (or the World Wide Web) to send and receive e-mail or for any other purpose, such as shopping, searching for information, or booking travel?”. In MHAS, digital exclusion at the individual level was not available, so another question was asked: “Do you have Internet access at home?”. For SHARE, digital exclusion was assessed by asking, “In the last 7 days, have you used the Internet at least once for e-mailing, searching for information, shopping, or any other purpose?”. Answers “no” (CHARLS, HRS, MHAS and SHARE) or “less than once a week” (SHARE) were classified as digital exclusion, while answers “yes” or at least once a week were counted as digital inclusion [9].

### Measurement of cognitive impairment

Cognitive impairment was assessed using three cognitive function tests: orientation, memory, and executive function. Participants were asked if they could remember the date of that day (day of week, month, and year in CHARLS, ELSA, HRS, and SHARE; day of month, month, and year in MHAS). The total score of the orientation dimension was four/three points, with one point for each item. For memory, the interviewer will read out a set of words (10 words for CHARLS, ELSA, HRS and SHARE, and 8 words for MHAS) at a slow and steady rate (about one word every two seconds). Participants were asked to recall as many words aloud as possible in any order (immediate word recall). After other questions and tests, the participants were again asked to recall as many words as possible (delayed word recall). The total memory score was 20/16 points, which was the sum of the immediate and delayed word recall scores, with one point for each word. For the executive task, participants were asked to answer “One hundred minus 7 equals what? And 7 from that? And 7 from that? And 7 from that? And 7 from that?” One point is added for each correct answer. For the executive task, the participants were asked to subtract seven from 100 five times in a row, adding one point for each success. The total score of cognitive function was defined as the sum of the scores of orientation (4/3 points), memory (20/16 points), and executive (5 points), resulting in 29/24 points. These tests have been shown to have high validity and reliability in the studies of others [16–18].

There is no consensus regarding the diagnostic criteria for cognitive impairment. In our study, we used aging-associated cognitive decline (AACD) to define cognitive impairment, namely at least one standard deviation (SD) below the age norm [19, 20]. All participants over the age of 60 were divided into five years groups. Participants in each age group who met the AACD criteria were classified as having cognitive impairments.

### Covariates

The covariates were determined through a literature review. Covariates that might confound the association between digital exclusion and cognitive impairment in the analyses included age [21], gender [22], education [21], labor force status [9], household wealth [23], married or partnered [21], co-residence with children [21], smoking [21], drinking [24], hypertension [22], stroke [22] cancer [22], and depressive symptoms [25)]. Further details on the covariates are presented in Supplementary Table S1.

### Statistical analysis

The baseline characteristics of CHARLS, ELSA, HRS, MHAS, and SHARE are described, respectively. For descriptive statistics, the mean ± SD was used for continuous variables and numbers and percentages for categorical variables.

**Fig. 2:**
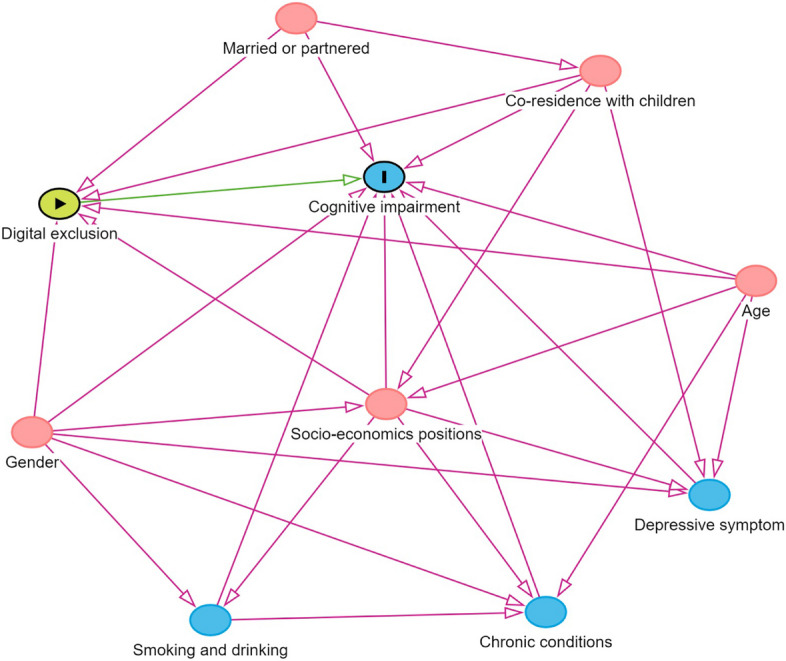
Causal directed acyclic graph. Note: The minimal sufficient adjustment set includes age, gender, socio-economic positions (education, labour force status, and household wealth), married or partnered and co-residence with children. The green arrow shows the main effect of interest

In order to solve the correlation problem of repeated measures in each cohort, the Generalized Estimation Equations (GEE) fitted binary logistic regression with exchangable correlation to investigate the relationship between digital exclusion and cognitive impairment, expressed as odds ratio (ORs) and 95% confidence intervals (CIs). While the association between scores of total cognitive function and three dimensions (orientation, memory, and executive) and digital exclusion was analyzed using a linear regression model, expressed in regression coefficients (β) and 95% CIs. We performed a univariate analysis between covariates and cognitive impairment and cognitive scores in each cohort to verify the reliability of identifying cognitive impairment. Then we used different confounding covariables in the three models to assess the robustness of the relationship between digital exclusion and cognitive impairment. Specifically, Model 1 did not adjust for any variables; Model 2 adjust the minimum sufficiently adjusted set (MSAS) as determined by the causally directed acyclic graph (DAGs) (Fig. 2), including age, gender, education, labour force status, household wealth, married or partnered and co-residence with children; Model 3 added smoking, drinking, hypertension, stroke, cancer, and depressive symptoms (the fully adjusted model).

In addition, stratified analysis was used to determine the relationship between cognitive impairment, scores of total cognition and three dimensions (orientation, memory, and executive), and digital exclusion in different age groups, gender, education, labor force status, household wealth, married or partnered status, co-residence with children, hypertension, stroke, cancer, and depressive symptoms.

To test the robustness of this study, we performed a sensitivity analysis. First of all, there are different degrees of data missing in each cohort, and the number and proportion of missing covariates are shown in Supplementary Table 2. We repeated the interpolated data through multiple interpolation of missing covariates to carry out the GEE model fitting binary logistic regression. Second, we repeated our analysis of the relationship between digital exclusion and cognitive impairment by excluding participants who had cognitive impairment at baseline. Multiple interpolation is implemented by the Template method (R Package “VIM”) [26] and multiple interpolation method (R Package “mice”) [27]. In order to avoid the bias caused by large heterogeneity, we used random effect model and forest plot to perform meta-analysis on the data of the five cohorts. All statistical analyses were conducted using R (version 4.1.0) and Empower ® software (http://www.empowerstats.net/cn/index.php), and the significance level was set at 0.05.

## Results

**Fig. 3:**
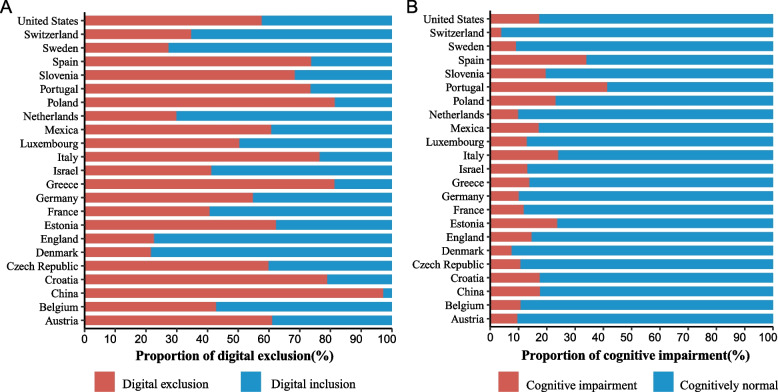
Distribution of digital exclusion (**A**) and cognitive impairment (**B**) of study participants by geographic

Table 1 presents the baseline characteristics of observations from the five longitudinal studies. The average age of the included participants for CHARLS, ELSA, HRS, MHAS, and SHARE was approximately 70 years, and the proportion of female participants ranged from 46.87% in CHARLS to 58.96% in HRS. The proportion of digital exclusion among older adults varies widely from country to country, from 21.69% in Denmark (SHARE) to 97.15% in China (CHARLS) (Fig. 3). There was little difference in the prevalence of cognitive impairment among older adults in different countries (Fig. 3). In addition, we described the characteristics of observations after multiple interpolation in Supplementary Table S3.

**Table 1:** Characteristics of the study participants

	**CHARLS (*N* = 16,698)**	**ELSA (*N* = 15,271)**	**HRS (*N* = 38,640)**	**MHAS (*N* = 17,385)**	**SHARE (*N* = 23,624)**
**Age**	67.35 ± 5.97	70.91 ± 7.48	75.30 ± 7.36	71.06 ± 7.59	70.52 ± 7.56
**Gender**					
** Male**	8872 (53.13%)	7267 (47.59%)	15,857 (41.04%)	7546 (43.41%)	10,757 (45.53%)
** Female**	7826 (46.87%)	8004 (52.41%)	22,783 (58.96%)	9839 (56.59%)	12,867 (54.47%)
**Education**					
**Less than upper secondary**	15,296 (91.60%)	3809 (24.94%)	8506 (22.01%)	15,419 (88.69%)	10,675 (45.19%)
** Upper secondary and vocational training**	1230 (7.37%)	8115 (53.14%)	13,210 (34.19%)	458 (2.63%)	7906 (33.47%)
** Tertiary**	172 (1.03%)	3347 (21.92%)	16,924 (43.80%)	1508 (8.67%)	5043 (21.35%)
**Labour force status**					
** Currently not working**	7594 (45.48%)	12,042 (78.86%)	31,413 (81.30%)	12,291 (70.70%)	19,852 (84.03%)
** Currently working without retirement**	8168 (48.92%)	2762 (18.09%)	3061 (7.92%)	5094 (29.30%)	2610 (11.05%)
**Currently working after retirement**	936 (5.61%)	467 (3.06%)	4166 (10.78%)	—^a^	1162 (4.92%)
**Household wealth**					
** Low tertile**	5331 (31.93%)	5089 (33.32%)	12,815 (33.17%)	5782 (33.26%)	7875 (33.33%)
** Medium tertile**	5769 (34.55%)	5089 (33.32%)	12,899 (33.38%)	5420 (31.18%)	7874 (33.33%)
** High tertile**	5598 (33.52%)	5093 (33.35%)	12,926 (33.45%)	6183 (35.57%)	7875 (33.33%)
** Married or partnered**	13,782 (82.54%)	10,805 (70.76%)	22,504 (58.24%)	11,131 (64.03%)	18,556 (78.55%)
** Co-residence with children**	7131 (42.71%)	113 (0.74%)	27,680 (71.64%)	11,952 (68.75%)	4028 (17.05%)
** Smoking**	4792 (28.70%)	1234 (8.08%)	3346 (8.66%)	1703 (9.80%)	3336 (14.12%)
** Drinking**	5613 (33.61%)	13,255 (86.80%)	19,056 (49.32%)	3879 (22.31%)	12,114 (51.28%)
** Hypertension**	6566 (39.32%)	7139 (46.75%)	26,339 (68.17%)	11,535 (66.35%)	11,709 (49.56%)
** Stroke**	911 (5.46%)	746 (4.89%)	4273 (11.06%)	816 (4.69%)	1383 (5.85%)
** Cancer**	255 (1.53%)	2183 (14.30%)	7733 (20.01%)	799 (4.60%)	2362 (10.00%)
** Depressive symptom**	5893 (35.29%)	2530 (16.57%)	7798 (20.18%)	5575 (32.07%)	6008 (25.43%)
** Digital exclusion**	16,222 (97.15%)	3453 (22.61%)	22,288 (57.68%)	10,567 (60.78%)	13,373 (56.61%)
** Cognitive impairment**	2939 (17.60%)	2237 (14.65%)	6692 (17.32%)	2990 (17.20%)	3591 (15.20%)
** Orientation scores**	2.99 ± 1.08	3.81 ± 0.49	3.64 ± 0.74	2.47 ± 0.88	3.83 ± 0.50
** Memory scores**	6.51 ± 3.51	10.84 ± 3.45	8.89 ± 3.49	8.47 ± 3.08	8.64 ± 3.49
** Executive scores**	3.47 ± 1.60	4.41 ± 0.97	3.32 ± 1.73	2.85 ± 1.61	4.17 ± 1.31
** Total cognitive scores**	12.96 ± 4.78	19.06 ± 3.90	15.84 ± 4.76	13.79 ± 4.26	16.63 ± 4.34

*CHARLS* China Health and Retirement Longitudinal Study, *ELSA*English Longitudinal Study of Ageing, *HRS* Health and Retirement Study, *MHAS* Mexican Health and Aging Study, *SHARE* Survey of Health, Ageing and Retirement in Europe

Continuous variables were expressed as mean ± standard deviation (SD) in case of normal distribution and categorical variables are presented as counts (percentages)

^a^For MHAS, the question on retirement was unavailable, so labour force status was recoded into currently working and currently not working

Supplementary Table S4-8 shows the univariate analysis results between covariates and cognitive impairment and cognitive score. The five cohort studies all show that higher education, current working, high level of household wealth, drinking status are associated with lower risk of cognitive impairment, and chronic disease and depressive symptoms are associated with higher risk of cognitive impairment.

Table 2 shows the association between digital exclusion and cognitive impairment, total cognitive scores, and three dimensions (orientation, memory, and executive). In the crude model (Model 1), digital exclusion was significantly associated with cognitive impairment, total cognitive scores, and three dimensions (orientation, memory, and executive). In the adjusted model (Model 3), those associations remained statistically significant in CHARLS (OR = 2.81, 95%CI [1.98,4.47] for cognitive impairment; β = -0.18, 95%CI [-0.26,-0.11] for orientation scores; β = -1.41, 95%CI [-1.74,-1.09] for memory scores; β = -0.31,95%CI [-0.41,-0.22] for executive scores; β = -1.90,95%CI [-2.29,-1.51] for total cognitive scores), ELSA (1.92[1.70,2.18]) for cognitive impairment; -0.08[-0.11,-0.05] for orientation scores; -0.87[-1.02,-0.72] for memory scores; -0.20[-0.25,-0.15] for executive scores; -1.13[-1.30,-0.96] for total cognitive scores), HRS (2.48[2.28,2.71)] for cognitive impairment; -0.11[-0.13,-0.09] for orientation scores; -0.96[-1.04,-0.87] for memory scores; -0.36[-0.40,-0.32] for executive scores; -1.35[-1.46,-1.24] for total cognitive scores), MHAS (1.92[1.74,2.12] for cognitive impairment; -0.20[-0.23,-0.17] for orientation scores; -0.73[-0.82,-0.64] for memory scores; -0.37[-0.42,-0.32] for executive scores; -1.20[-1.33,-1.08] for total cognitive scores), SHARE (2.60[2.34,2.88] for cognitive impairment; -0.03[-0.05,-0.02] for orientation scores; -1.19[-1.28,-1.10] for memory scores; -0.29[-0.33,-0.26] for executive scores; -1.51[-1.62,-1.41] for total cognitive scores).

**Table 2:** Associations between digital exclusion and cognitive impairment

		**CHARLS**	**ELSA**	**HRS**	**MHAS**	**SHARE**
		**OR/β (95% CI) *P* value**	**OR/β (95% CI) *P* value**	**OR/β (95% CI) *P* value**	**OR/β (95% CI) *P* value**	**OR/β (95% CI) *P* value**
**Cognitive impairment**	**Model 1**	4.57 (3.25, 6.43) < 0.001	2.73 (2.46, 3.04) < 0.001	4.17 (3.87, 4.49) < 0.001	2.39 (2.18, 2.63) < 0.001	4.12 (3.77, 4.51) < 0.001
	**Model 2**	2.98 (1.98, 4.47) < 0.001	2.03 (1.79, 2.30) < 0.001	2.64 (2.42, 2.87) < 0.001	1.98 (1.80, 2.18) < 0.001	2.75 (2.49, 3.05) < 0.001
	**Model 3**	2.81 (1.84, 4.28) < 0.001	1.92 (1.70, 2.18) < 0.001	2.48 (2.28, 2.71) < 0.001	1.92 (1.74, 2.12) < 0.001	2.60 (2.34, 2.88) < 0.001
**Orientation scores**	**Model 1**	-0.49 (-0.57, -0.42) < 0.001	-0.14 (-0.17, -0.12) < 0.001	-0.26 (-0.27, -0.24) < 0.001	-0.28 (-0.31, -0.26) < 0.001	-0.12 (-0.13, -0.10) < 0.001
	**Model 2**	-0.21 (-0.28, -0.13) < 0.001	-0.08 (-0.11, -0.06) < 0.001	-0.12 (-0.14, -0.11) < 0.001	-0.21 (-0.24, -0.18) < 0.001	-0.04 (-0.05, -0.03) < 0.001
	**Model 3**	-0.18 (-0.26, -0.11) < 0.001	-0.08 (-0.11, -0.05) < 0.001	-0.11 (-0.13, -0.09) < 0.001	-0.20 (-0.23, -0.17) < 0.001	-0.03 (-0.05, -0.02) < 0.001
**Memory scores**	**Model 1**	-2.63 (-2.97, -2.29) < 0.001	-2.23 (-2.39, -2.08) < 0.001	-2.08 (-2.17, -2.00) < 0.001	-1.16 (-1.26, -1.07) < 0.001	-2.59 (-2.67, -2.50) < 0.001
	**Model 2**	-1.53 (-1.87, -1.20) < 0.001	-0.94 (-1.09, -0.79) < 0.001	-1.04 (-1.12, -0.96) < 0.001	-0.77 (-0.86, -0.68) < 0.001	-1.29 (-1.39, -1.20) < 0.001
	**Model 3**	-1.41 (-1.74, -1.09) < 0.001	-0.87 (-1.02, -0.72) < 0.001	-0.96 (-1.04, -0.87) < 0.001	-0.73 (-0.82, -0.64) < 0.001	-1.19 (-1.28, -1.10) < 0.001
**Executive scores**	**Model 1**	-0.77 (-0.85, -0.68) < 0.001	-0.38 (-0.43, -0.33) < 0.001	-0.76 (-0.80, -0.72) < 0.001	-0.58 (-0.63, -0.53) < 0.001	-0.70 (-0.73, -0.67) < 0.001
	**Model 2**	-0.35 (-0.45, -0.25) < 0.001	-0.21 (-0.26, -0.16) < 0.001	-0.39 (-0.43, -0.35) < 0.001	-0.38 (-0.44, -0.33) < 0.001	-0.33 (-0.36, -0.29) < 0.001
	**Model 3**	-0.31 (-0.41, -0.22) < 0.001	-0.20 (-0.25, -0.15) < 0.001	-0.36 (-0.40, -0.32) < 0.001	-0.37 (-0.42, -0.32) < 0.001	-0.29 (-0.33, -0.26) < 0.001
**Total cognitive scores**	**Model 1**	-3.84 (-4.25, -3.43) < 0.001	-2.73 (-2.91, -2.54) < 0.001	-2.96 (-3.07, -2.85) < 0.001	-1.85 (-1.98, -1.72) < 0.001	-3.40 (-3.50, -3.30) < 0.001
	**Model 2**	-2.07 (-2.47, -1.67) < 0.001	-1.22 (-1.39, -1.04) < 0.001	-1.46 (-1.57, -1.35) < 0.001	-1.26 (-1.38, -1.13) < 0.001	-1.66 (-1.77, -1.55) < 0.001
	**Model 3**	-1.90 (-2.29, -1.51) < 0.001	-1.13 (-1.30, -0.96) < 0.001	-1.35 (-1.46, -1.24) < 0.001	-1.20 (-1.33, -1.08) < 0.001	-1.51 (-1.62, -1.41) < 0.001

*CHARLS* China Health and Retirement Longitudinal Study, *ELSA* English Longitudinal Study of Ageing, *HRS* Health and Retirement Study, *MHAS* Mexican Health and Aging Study, *SHARE* Survey of Health, Ageing and Retirement in Europe

Model 1: No variables are adjusted

Model 2: Adjusted for the minimal sufficient adjustment set (MSAS) identified using a causal directed acyclic graph (DAG) including further adjusted for age, gender, education, labour force status, household wealth, married or partnered and co-residence with children

Model 3: Further adjusted for smoking, drinking, hypertension, stroke, cancer, and depressive symptom based on Model 2

To assess the heterogeneity of digital exclusion on cognitive impairment, Table 3 presents the digital exclusion on cognitive impairment. In addition, the heterogeneity of digital exclusion on the scores of the three dimensions (orientation, memory, and executive) and total cognition in different subpopulations is presented in Supplementary Table S9-12.

**Table 3:** Association between digital exclusion and cognitive impairment by age, gender, education, labour force status, household wealth, married or partnered, co-residence with children, smoking, drinking, hypertension, stroke, cancer and depressive symptoms

Subgroups	CHARLS		ELSA		HRS		MHAS		SHARE	
	**OR (95% CI)**	***P* for interaction**	**OR (95% CI)**	***P* for interaction**	**OR (95% CI)**	***P* for interaction**	**OR (95% CI)**	***P* for interaction**	**OR (95% CI)**	***P* for interaction**
**Age**		0.532		0.039		0.056		0.008		0.006
** 60–79**	2.77 (1.82, 4.22)		1.79 (1.56, 2.06)		2.48 (2.25, 2.73)		2.02 (1.82, 2.25)		2.40 (2.16, 2.66)	
** ≥ 80**	—^a^		—^a^		3.07 (2.49, 3.79)		1.46 (1.13, 1.90)		5.25 (2.88, 9.55)	
**Gender**		0.830		0.416		0.023		0.968		0.075
** Male**	3.36 (1.76, 6.40)		1.81 (1.53, 2.16)		2.32 (2.04, 2.65)		1.92 (1.65, 2.22)		2.39 (2.07, 2.76)	
** Female**	2.56 (1.43, 4.58)		2.05 (1.71, 2.45)		2.62 (2.32, 2.94)		1.92 (1.68, 2.18)		2.88 (2.48, 3.36)	
**Education**		0.920		0.933		0.002		0.043		0.243
** Less than upper secondary**	3.00 (1.97, 4.57)		1.93 (1.61, 2.32)		1.98 (1.67, 2.34)		1.96 (1.78, 2.17)		2.74 (2.37, 3.17)	
** Upper secondary and vocational training**	2.70 (0.62, 11.85)		1.98 (1.66, 2.36)		2.27 (1.98, 2.60)		0.57 (0.14, 2.37)		2.33 (1.97, 2.76)	
** Tertiary**	—^a^		—^a^		2.89 (2.51, 3.33)		1.13 (0.45, 2.80)		2.98 (2.21, 4.00)	
**Labour force status**		0.106		0.210		0.053		0.640		0.747
** Currently not working**	3.61 (1.92, 6.81)		1.97 (1.73, 2.24)		2.52 (2.30, 2.77)		1.89 (1.69, 2.12)		2.57 (2.29, 2.87)	
** Currently working without retirement**	1.49 (0.77, 2.89)		1.47 (0.96, 2.25)		1.97 (1.41, 2.74)		2.10 (1.73, 2.55)		2.76 (2.05, 3.72)	
** Currently working after retirement**	—^a^		3.04 (1.22, 7.55)		2.59 (1.95, 3.45)		—^b^		3.03 (1.86, 4.91)	
**Household wealth**		0.686		0.999		0.189		0.328		0.009
** Low tertile**	3.73 (1.55, 8.98)		1.95 (1.64, 2.31)		2.45 (2.15, 2.79)		2.15 (1.81, 2.56)		2.15 (1.81, 2.55)	
** Medium tertile**	3.12 (1.31, 7.47)		1.98 (1.60, 2.46)		2.35 (2.03, 2.71)		1.91 (1.60, 2.28)		2.72 (2.29, 3.23)	
** High tertile**	2.50 (1.36, 4.57)		1.95 (1.46, 2.60)		2.81 (2.34, 3.36)		1.74 (1.49, 2.04)		3.21 (2.64, 3.90)	
**Married or partnered**		0.867		0.611		0.697		0.023		0.014
** No**	3.41 (0.94, 12.32)		2.01 (1.63, 2.48)		2.47 (2.16, 2.82)		1.69 (1.44, 1.99)		3.56 (2.71, 4.68)	
** Yes**	2.83 (1.81, 4.41)		1.89 (1.62, 2.20)		—#		2.11 (1.87, 2.38)		2.49 (2.22, 2.78)	
**Co-residence with children**		0.121		0.343		0.707		0.031		0.936
** No**	2.19 (1.29, 3.73)		1.92 (1.70, 2.18)		—^a^		2.33 (1.87, 2.91)		2.60 (2.32, 2.91)	
** Yes**	5.93 (2.09, 16.79)		0.58 (0.08, 4.17)		2.55 (2.30, 2.82)		1.84 (1.65, 2.05)		2.63 (2.04, 3.38)	
**Smoking**		0.175		0.092		0.063		0.933		0.336
** No**	2.35 (1.43, 3.86)		1.99 (1.74, 2.27)		2.53 (2.31, 2.78)		1.90 (1.72, 2.10)		2.65 (2.37, 2.97)	
** Yes**	6.24 (2.08, 18.72)		1.55 (1.09, 2.20)		2.22 (1.71, 2.87)		2.07 (1.48, 2.90)		2.32 (1.81, 2.98)	
**Drinking**		0.126		0.085		0.743		0.031		0.002
** No**	2.19 (1.21, 3.96)		2.28 (1.76, 2.97)		2.38 (2.12, 2.67)		1.83 (1.64, 2.04)		2.21 (1.92, 2.55)	
** Yes**	5.25 (2.15, 12.84)		1.86 (1.61, 2.14)		2.69 (2.36, 3.07)		2.50 (2.00, 3.11)		3.08 (2.65, 3.57)	
**Hypertension**		0.701		0.563		0.002		< 0.001		0.201
** No**	3.20 (1.82, 5.61)		1.96 (1.63, 2.36)		2.99 (2.54, 3.52)		2.47 (2.06, 2.96)		2.44 (2.12, 2.80)	
** Yes**	2.62 (1.34, 5.11)		1.91 (1.61, 2.25)		2.31 (2.09, 2.56)		1.73 (1.54, 1.94)		2.79 (2.39, 3.25)	
**Stroke**		0.044		0.568		0.034		0.004		0.171
** No**	2.55 (1.66, 3.93)		1.91 (1.67, 2.17)		2.56 (2.33, 2.82)		2.01 (1.81, 2.22)		2.55 (2.29, 2.83)	
** Yes**	—^a^		2.18 (1.42, 3.34)		2.17 (1.74, 2.72)		1.15 (0.80, 1.66)		3.39 (2.27, 5.07)	
**Cancer**		0.416		0.179		0.067		0.967		0.493
** No**	2.91 (1.94, 4.37)		1.87 (1.64, 2.15)		2.60 (2.36, 2.86)		1.92 (1.74, 2.12)		2.64 (2.37, 2.94)	
** Yes**	1.26 (0.20, 8.04)		2.36 (1.71, 3.26)		2.15 (1.77, 2.61)		2.08 (1.26, 3.43)		2.33 (1.68, 3.25)	
**Depressive symptom**		0.471		0.163		< 0.001		0.385		0.184
** No**	3.21 (1.95, 5.28)		1.84 (1.60, 2.13)		2.75 (2.48, 3.04)		2.04 (1.81, 2.30)		2.50 (2.21, 2.82)	
** Yes**	2.26 (0.98, 5.19)		2.35 (1.84, 2.98)		2.02 (1.71, 2.39)		1.86 (1.58, 2.19)		2.92 (2.40, 3.54)	

*CHARLS* China Health and Retirement Longitudinal Study, *ELSA* English Longitudinal Study of Ageing, *HRS* Health and Retirement Study, *MHAS* Mexican Health and Aging Study, *SHARE* Survey of Health, Ageing and Retirement in Europe

In addition to the stratification variables themselves, age, gender, education, labour force status, household wealth, married or partnered, co-residence with children, smoking, drinking, hypertension, stroke, cancer and depressive symptoms

^a^The model failed because of the small sample size

^b^For MHAS, the question on retirement was unavailable, so labour force status was recoded into currently working and currently not working

In sensitivity analysis, we repeated GEE model analysis with data after multiple interpolation, and the association was statistically significant in all five cohort studies (Supplementary Table S13) and remained statistically significant after excluding participants with cognitive impairment at baseline (Supplementary Table S14).

Figure S1 shows the results of meta-analysis. After meta-analysis of 5 cohorts based on the random effects model, the correlation OR value between digital rejection and cognitive dysfunction in the elderly was 2.26 (95%Cl: 1.94–2.62).

## Discussion

We investigated the relationship between digital exclusion and cognitive impairment in older adults in five longitudinal studies. Digital exclusion accounts for 21.69% in Denmark, 22.61% in the United Kingdom, 60.78% in Mexico and 97.15% in China. After adjusting for potential covariates, digital exclusion was positively associated with cognitive impairment.

Although previous studies have described the relationship between digital exclusion and cognitive impairment [10], this study was only conducted in an elderly Chinese population and does not necessarily apply to older adults globally. Our study is one of the first to examine the relationship between digital exclusion and cognitive impairment in a global sample of older adults. Over the past decade, as an increasing number of people have been exposed to the Internet, researchers in the field of health have been quick to use it to promote health management [28, 29]. However, the rate of digital exclusion among the elderly population remains relatively high, especially in China, which limits the potential of the Internet as a platform to achieve better health management. Based on older people in multiple countries, we were able to investigate whether digital exclusion has the same effect on cognitive impairment in older people and, more importantly, which groups in the older population are more sensitive to digital exclusion in terms of cognitive impairment.

By including five longitudinal studies from 23 countries, digital exclusion was found to be associated with cognitive impairment in older populations. These findings are in line with those of previous studies that have shown a reduced risk of cognitive impairment among older adults who use the Internet [30–32]. However, few studies have investigated whether this effect is valid in older adults. This study found that digital exclusion was positively associated with cognitive impairment in older adults in the CHARLS, ELSA, HRS, MHAS, and SHARE groups, and this association was more significant in the CHARLS,SHARE and HRS groups. This may be due to a much higher proportion of participants in the CHARLS being digitally excluded than in other cohorts; The average age of HRS participants was relatively higher than that of other cohorts; Participants with cognitive impairment were higher in SHARE. The number and proportion of participants with digital exclusion and cognitive impairment were lower in ELSA. Alcohol consumption was relatively low among participants in MHAS, and alcohol consumption is a high-risk factor for cognitive impairment in older adults [24, 33, 34]. We also evaluated the relationship between digital exclusion and the three dimensions (orientation, memory, and executive) of cognitive impairment and found that digital exclusion was negatively correlated with these three dimensions. This has not been reported in previous studies and it is hoped that future studies will confirm this finding.

In the subgroup analysis, the relationship between digital exclusion and cognitive impairment was not found to be more significant in any population. Previous studies have suggested that digital exclusion is more strongly associated with cognitive impairment in people over the age of 80 [10], but this study did not draw such a conclusion, possibly due to the small sample size of people > 80 years of age or confounding bias due to unmeasured covariates; therefore, follow-up studies are needed to confirm this. We also performed a subgroup analysis of digital exclusion and the three dimensions of cognitive impairment but did not find which population had a stronger relationship between digital exclusion and cognitive impairment.

We also explore the potential mechanisms underlying the relationship between digital exclusion and cognitive impairment. First, older adults who are not exposed to the Internet are more likely to experience social isolation, loneliness, and depression due to digital exclusion. Social isolation, loneliness, and depression directly affect cardiovascular function, and also affect cognitive function by reducing cerebral blood flow and inhibiting neurovascular coupling, leading to significant impairments in different cognitive dimensions such as memory, computation, orientation, and so on, leading to cognitive impairment [35, 36]. Older adults with access to the Internet are more likely to obtain more information and communication resources from the network environment, for example, older people can obtain and understand the latest health management information through the Internet, purchase medicines and health products, and the Internet also provides opportunities for older people to consult health professionals in a timely manner and real-time data monitoring, thus obtaining greater cognitive benefits and improving cognitive function [9]. Digital exclusion was associated with cognitive impairment and all three dimensions of cognitive impairment, suggesting that it is a potential risk factor for overall healthy functioning in older adults [10]. Second, Internet use may impact cognitive function by affecting pallidum volume. Previous research has shown that Internet users have a larger pallidum volume, with the volume on the left being positively associated with changes in MMSE scores, suggesting that pallidum volume may be a protective factor against cognitive decline [31].

The current findings suggest that Internet use can reduce the incidence of cognitive impairment in older adults, highlighting that digital inclusion may be an important strategy to improve cognitive function and reduce the risk of cognitive impairment in older adults. In the spread of COVID-19, the general cognitive ability of patients recovering from COVID-19 is lower than that of healthy control group [37, 38]. Due to the inconvenience of face-to-face communication, the incidence of cognitive impairment in the elderly is also increased [39]. There is ample evidence that the Internet profoundly changes people’s thoughts and behaviors [40, 41]. Building a digitally inclusive society through ICT training [36] and the use of smartphone technology [42] in the aging process can helps people achieve early, timely, and long-term health management in their later years [9]. Therefore, further research is needed on Internet-based interventions to address cognitive impairment in the elderly population. In addition to Internet-based interventions, interventions such as cognitive training, the Mediterranean diet, and physical activity have also been shown to be positively associated with cognitive outcomes and may also be key to maintaining cognitive health and delaying cognitive decline [43–45].

This study has two advantages. First, the sample included five longitudinal study from 23 countries across three continents, and the large sample size increased the reliability and robustness of the statistical analysis. Second, all participants were recruited from a large, representative national sample, and the five surveys were standardized to allow comparisons across the five databases. However, our study had certain limitations. First, because this was an observational cross-sectional study, no causal relationship could be established. Further experimental studies are needed to infer a causal relationship between digital exclusion and cognitive impairment. Second, there is information bias due to different exposure measures, such as individual-level Internet use in CHARLS, ELSA, HRS, and SHARE and home-level Internet access in MHAS, which may reduce the comparability of the five cohorts. Third, owing to differences in the assessment of cognitive function across the five longitudinal studies, the year of final inclusion differed to achieve uniformity of cognitive function. Fourth, although various confounding factors were considered, unmeasured covariates may have led to confounding biases. Fifth, we cannot rule out the possibility of an inverse association between the number of rejections and cognitive impairment.

## Conclusion

Our research shows that a significant proportion of older people suffer from digital exclusion, especially in China. Digital exclusion was positively correlated with cognitive impairment. These findings suggest that digital inclusion could be an important strategy to improve cognitive function and reduce the risk of cognitive impairment in older adults.

### Supplementary Information

**Supplementary Material 1.**

## Data Availability

The original survey datasets from CHARLS, ELSA, HRS, MHAS and SHARE are all free of charge and available from the Global Ageing Data Portal (https://g2aging.org/).
