# Correction: Digital exclusion and cognitive impairment in older people: findings from five longitudinal studies

**DOI:** 10.1186/s12877-024-05121-y

**Published:** 2024-06-21

**Authors:** Yuge Wang, Zhigang Wu, Lanzhi Duan, Sijia Liu, Ruzhao Chen, Tao Sun, Jiang Wang, Jianghua Zhou, Hongxia Wang, Pan Huang

**Affiliations:** 1 College of Nursing, Wenzhou Medical University, Wenzhou, China; 2 Department of Epidemiology and Health Statistics, Xiangya School of Public Health, Central South University, Changsha, Hunan China; 3 Center for Clinical Medicine Researchof, JingGangshan University, Affiliated Hospital of JingGangshan University, Ji’an, China; 4 Department of Medicine, JingGangshan University, Ji’an, China; 5 Online Collaborative Research Center for Evidence-Based Medicine, Ministry of Education, JingGangshan University, Ji’an, China; 6 Department of Cardiology, The First Affiliated Hospital of Wenzhou Medical University, Wenzhou, China; 7 Department of Nursing, The First Affiliated Hospital of Wenzhou Medical University, Wenzhou, China

**Correction** **:** **BMC Geriatr 24, 406 (2024)**

**https://doi.org/10.1186/s12877-024-05026-w**

After publication of this article [1], the authors reported that a wrong Supplementary file was originally published with this article; it has now been replaced with the correct file. The Supplementary Material 1 that contained crude data has been removed, and the Supplementary Material that includes a brief introduction to the five cohorts, Tables S1-S14, Figure S1, and the STROBE Statement has been added.

The original article has been corrected.
