# Supplementary material for: Digital exclusion and cognitive impairment in older people: findings from five longitudinal studies
Source: BMC Geriatr. 2024 May 7;24:406. doi: 10.1186/s12877-024-05026-w (PMC11077883; doi:10.1186/s12877-024-05026-w)
Supplement: Supplementary file 1 — Supplementary Material 1. [file 12877_2024_5026_MOESM1_ESM.docx]

**supplementary materials**

**A brief introduction to the five cohorts**

**CHARLS:** The purpose of the China Health and Retirement Longitudinal Study (CHARLS) is to collect a set of high-quality microdata representing families and individuals of middle-aged and elderly people aged 45 years and older in China, so as to analyze the aging problem of the Chinese population and promote interdisciplinary research on the aging problem. The survey covers 150 county-level units, 450 village-level units, and about 17,000 people in 10,000 families. The survey included basic demographic information of respondents and their families, transfer payments between family members, respondents' health status, medical care and insurance, employment, income, expenditure, and assets. In addition, CHARLS includes 13 body measurements and blood sampling. CHARLS has published four waves of data for this article, including a national baseline survey (wave 1, 2011), a first follow-up survey (wave 2, 2013), a second follow-up survey (wave 3, 2015), and a third follow-up survey (wave 4, 2018). CHARLS related data can be downloaded at the CHARLS home page at [https://charls.pku.edu.cn/ (1).](https://charls.pku.edu.cn/Obtain)

**ELSA:** The English Longitudinal Study of Ageing (ELSA) is a prospective cohort study involving the health, social, welfare and economic environmental dynamics of the UK population aged 50 years and older. Included cohort studies included wave 7 (2014 - 2015), wave 8 (2016 - 2017), and wave 9 (2018 - 2019). For more information about ELSA, visit <http://www.elsa-project.ac.uk/> (2).

**HRS:** Health and Retirement Study (HRS, <https://hrs.isr.umich.edu/> ), a nationally representative longitudinal study of older adults that included detailed economic and health information, surveyed a representative sample of approximately 20,000 people in the United States. The survey has been followed up every 2 years since 1992 until 2020 (3,4). Cohort studies included in the HRS included wave 10 (2010 - 2011), wave 11 (2012 - 2013), wave 12 (2014 - 2015), and wave 13 (2016 - 2017).

**MHAS:** Mexican Health and Aging Study (MHAS, <https://www.mhasweb.org/> ), a nationwide longitudinal study of adults aged 50 years and older in Mexico. The baseline survey was conducted in 2001 and covered adults born 1951 or earlier across the country and urban/rural areas, with follow-up surveys conducted in 2003, 2012, 2015, 2018, and 2021.

**SHARE:** The Survey of Health, Ageing and Retirement in Europe (SHARE, <https://share-eric.eu/> ), is a study used to investigate the impact of health, social, economic and environmental policies in the life course of European citizens. From 2004 to the present, 530,000 in-depth interviews have been conducted with 140,000 people over the age of 50 from 28 European countries and Israel. Participants were followed every 2 years from wave 1 (2004 - 2006) to wave 7 (2017 - 2019)(5–7).

Reference

1. Hu Y, Peng W, Ren R, Wang Y, Wang G. Sarcopenia and mild cognitive impairment among elderly adults: The first longitudinal evidence from CHARLS. J Cachexia Sarcopenia Muscle. 2022 Dec;13(6):2944–52.

2. Na-Ek N, Srithong J, Aonkhum A, Boonsom S, Charoen P, Demakakos P. Educational level as a cause of type 2 diabetes mellitus: Caution from triangulation of observational and genetic evidence. Acta Diabetol. 2022 Jan;59(1):127–35.

3. Ellwardt L, Hank K, Mendes de Leon CF. Grandparenthood and risk of mortality: Findings from the Health and Retirement Study. Soc Sci Med. 2021 Jan;268:113371.

4. Sonnega A, Faul JD, Ofstedal MB, Langa KM, Phillips JWR, Weir DR. Cohort Profile: the Health and Retirement Study (HRS). Int J Epidemiol. 2014 Apr;43(2):576–85.

5. Börsch-Supan A, Brandt M, Hunkler C, Kneip T, Korbmacher J, Malter F, et al. Data Resource Profile: the Survey of Health, Ageing and Retirement in Europe (SHARE). Int J Epidemiol. 2013 Aug;42(4):992–1001.

6. Tan X, Lebedeva A, Åkerstedt T, Wang HX. Sleep Mediates the Association Between Stress at Work and Incident Dementia: Study From the Survey of Health, Ageing and Retirement in Europe. J Gerontol A Biol Sci Med Sci. 2023 Mar 1;78(3):447–53.

7. Feng MY, Bi YH, Wang HX, Pei JJ. Influence of chronic diseases on the occurrence of depression: A 13-year follow-up study from the Survey of Health, Ageing and Retirement in Europe. Psychiatry Res. 2023 Aug;326:115268.

| **Supplementary Table S1 Descriptions of covariates** | |
| --- | --- |
| **Variables** |  |
| **Age** | The age was calculated from the date of interview and the date of birth. |
| **Gender** | The gender was reported as either male or female. |
| **Education** | According to the International Standard Classification of Education (ISCED) 1997, education is classified as lower secondary, upper secondary and vocational training, and tertiary. |
| **Labour force status** | With questions about current working status and self-reported retirement, the labor force status was coded as currently not working, currently working without retirement, and currently working after retirement. |
| **Household wealth** | The level of household wealth has been divided into tertiles of non-housing financial wealth. |
| **Married or partnered** | Marital status was classified as married/partnered or single, single covered separated, divorced, widowed, and never married. |
| **Co-residence with children** | Co-residence was viewed as a binary question, and no children missing was viewed as no co-residence. |
| **Smoking** | Smoking is described as current smoking behavior. |
| **Drinking** | Drinking is about whether alcohol was consumed last week, last year, or earlier. |
| **Hypertension** | Hypertensive are defined by whether or not the respondent had been advised by a doctor that they have or are currently suffering from hyperttensive. |
| **Stroke** | Stroke are defined by whether or not the respondent had been advised by a doctor that they have or are currently suffering from stroke. |
| **Cancer** | Cancer are defined by whether or not the respondent had been advised by a doctor that they have or are currently suffering from cancer. |
| **Depressive symptom** | We measured depressive symptoms using the Centre for Epidemiologic Studies of Depression (CES-D) scale in CHARLS (CESD-10), ELSA (CESD-8), HRS (CESD-8), MHAS (CESD-9) and Euro-D in SHARE with a score range of0-30, 0-8, 0-8, 0-12, and 0-12, respectively. Participants with a scores equal to or greater than the cutoff score (CHARLS≥10, ELSA≥3, HRS≥3, MHAS≥4 and SHARE≥5) was coded as 1, others were coded as 0. |

Notes: CHARLS: China Health and Retirement Longitudinal Study; ELSA: English Longitudinal Study of Ageing; HRS: Health and Retirement Study; MHAS: Mexican Health and Aging Study; SHARE: Survey of Health, Ageing and Retirement in Europe.

| **Supplementary Table S2 Missing number for covariates** | | | | | |
| --- | --- | --- | --- | --- | --- |
| **Variables** | **CHARLS** | **ELSA** | **HRS** | **MHAS** | **SHARE** |
|  | **Missing number (%)** | **Missing number (%)** | **Missing number (%)** | **Missing number (%)** | **Missing number (%)** |
| **Age** | 0 (0) | 0 (0) | 0 (0) | 0 (0) | 0 (0) |
| **Gender** | 0 (0) | 0 (0) | 0 (0) | 0 (0) | 0 (0) |
| **Education** | 205 (0.87%) | 1426 (7.81%) | 46 (0.12%) | 195 (1.05%) | 0 (0) |
| **Labour force status** | 311 (1.33%) | 1 (0.01%) | 63 (0.16%) | 54 (0.29%) | 624 (0.88%) |
| **Household wealth** | 4848 (20.67%) | 220 (1.21%) | 0 (0) | 366 (1.96%) | 0 (0) |
| **Married or partnered** | 0 (0) | 4 (0.02%) | 36 (0.09%) | 0 (0) | 62 (0.09%) |
| **Co-residence with children** | 339 (1.45%) | 0 (0) | 0 (0) | 987 (5.30%) | 7863 (11.09%) |
| **Smoking** | 1386 (5.91%) | 26 (0.14%) | 289 (0.74%) | 8 (0.04%) | 43325 (61.11%) |
| **Drinking** | 8 (0.03%) | 1482 (8.12%) | 11 (0.03%) | 3 (0.02%) | 9 (0.01%) |
| **Hypertension** | 657 (2.80%) | 0 (0) | 0 (0) | 15 (0.08%) | 18 (0.03%) |
| **Stroke** | 621 (2.65%) | 0 (0) | 0 (0) | 13 (0.07%) | 20 (0.03%) |
| **Cancer** | 657 (2.80%) | 0 (0) | 0 (0) | 15 (0.08%) | 17 (0.02%) |
| **Depressive symptom** | 15 (0.06%) | 19 (0.10%) | 10 (0.03%) | 10 (0.05%) | 0 (0) |

Notes: CHARLS: China Health and Retirement Longitudinal Study; ELSA: English Longitudinal Study of Ageing; HRS: Health and Retirement Study; MHAS: Mexican Health and Aging Study; SHARE: Survey of Health, Ageing and Retirement in Europe.

| **Supplementary Table S3 Characteristics of the study participants (The missing covariates are interpolated)** | | | | | |
| --- | --- | --- | --- | --- | --- |
|  | **CHARLS (N=23,459)** | **ELSA (N=18,254)** | **HRS (N=39,088)** | **MHAS (N=18,626)** | **SHARE (N=70,896)** |
| **Age** | 67.44 ± 6.05 | 71.18 ± 7.69 | 75.29 ± 7.36 | 70.99 ± 7.62 | 71.00 ± 7.80 |
| **Gender** |  |  |  |  |  |
| **Male** | 13,124 (55.94%) | 8,422 (46.14%) | 16,056 (41.08%) | 8,230 (44.19%) | 32,613 (46.00%) |
| **Female** | 10,335 (44.06%) | 9,832 (53.86%) | 23,032 (58.92%) | 10,396 (55.81%) | 38,283 (54.00%) |
| **Education** |  |  |  |  |  |
| **Less than upper secondary** | 21,192 (90.34%) | 4,402 (24.12%) | 8,627 (22.07%) | 16,469 (88.42%) | 30,244 (42.66%) |
| **Upper secondary and vocational training** | 1,951 (8.32%) | 9,093 (49.81%) | 13,337 (34.12%) | 495 (2.66%) | 25,217 (35.57%) |
| **Tertiary** | 316 (1.35%) | 4,759 (26.07%) | 1,7124 (43.81%) | 1,662 (8.92%) | 15,435 (21.77%) |
| **Labour force status** |  |  |  |  |  |
| **Currently not working** | 11,202 (47.75%) | 14,459 (79.21%) | 31,778 (81.30%) | 13,036 (69.99%) | 57,476 (81.07%) |
| **Currently working without retirement** | 10,889 (46.42%) | 3,265 (17.89%) | 3,103 (7.94%) | 5,590 (30.01%) | 8,269 (11.66%) |
| **Currently working after retirement** | 1,368 (5.83%) | 530 (2.90%) | 4,207 (10.76%) | —* | 5,151 (7.27%) |
| **Household wealth** |  |  |  |  |  |
| **Low tertile** | 6,908 (29.45%) | 6,084 (33.33%) | 12,979 (33.20%) | 6,380 (34.25%) | 23,406 (33.01%) |
| **Medium tertile** | 8,345 (35.57%) | 6,097 (33.40%) | 13,057 (33.40%) | 6,357 (34.13%) | 23,858 (33.65%) |
| **High tertile** | 8,206 (34.98%) | 6,073 (33.27%) | 13,052 (33.39%) | 5,889 (31.62%) | 23,632 (33.33%) |
| **Married or partnered** | 19,414 (82.76%) | 12,596 (69.00%) | 22,758 (58.22%) | 11,760 (63.14%) | 51,853 (73.14%) |
| **Co-residence with children** | 9,939 (42.37%) | 147 (0.81%) | 27,979 (71.58%) | 12,919 (69.36%) | 12,136 (17.12%) |
| **Smoking** | 10,359 (14.61%) | 10,359 (14.61%) | 10,359 (14.61%) | 10,359 (14.61%) | 10,359 (14.61%) |
| **Drinking** | 8,201 (34.96%) | 15,651 (85.74%) | 19,282 (49.33%) | 4,190 (22.50%) | 37,982 (53.57%) |
| **Hypertension** | 9,327 (39.76%) | 8,571 (46.95%) | 26,643 (68.16%) | 12,227 (65.64%) | 38,653 (54.52%) |
| **Stroke** | 1,365 (5.82%) | 927 (5.08%) | 4,319 (11.05%) | 856 (4.60%) | 5,186 (7.31%) |
| **Cancer** | 673 (2.87%) | 2,630 (14.41%) | 7,818 (20.00%) | 848 (4.55%) | 7,980 (11.26%) |
| **Depressive symptom** | 7,902 (33.68%) | 3,214 (17.61%) | 7,897 (20.20%) | 5,937 (31.87%) | 18,306 (25.82%) |
| **Digital exclusion** | 22,492 (95.88%) | 5,205 (28.51%) | 22,556 (57.71%) | 11,330 (60.83%) | 39,016 (55.03%) |
| **Cognitive impairment** | 3,973 (16.94%) | 2,800 (15.34%) | 6,782 (17.35%) | 3,125 (16.78%) | 10,610 (14.97%) |
| **Orientation scores** | 3.04 ± 1.07 | 3.79 ± 0.52 | 3.64 ± 0.74 | 2.47 ± 0.87 | 3.83 ± 0.50 |
| **Memory scores** | 6.64 ± 3.52 | 10.65 ± 3.53 | 8.89 ± 3.50 | 8.47 ± 3.08 | 8.82 ± 3.58 |
| **Executive scores** | 3.53 ± 1.58 | 4.38 ± 1.00 | 3.31 ± 1.73 | 2.86 ± 1.61 | 4.18 ± 1.30 |
| **Total cognitive scores** | 13.21 ± 4.79 | 18.82 ± 4.04 | 15.84 ± 4.76 | 13.80 ± 4.27 | 16.84 ± 4.41 |

Notes: CHARLS: China Health and Retirement Longitudinal Study; ELSA: English Longitudinal Study of Ageing; HRS: Health and Retirement Study; MHAS: Mexican Health and Aging Study; SHARE: Survey of Health, Ageing and Retirement in Europe.

Continuous variables were expressed as mean±standard deviation (SD) in case of normal distribution and categorical variables are presented as counts (percentages).

*For MHAS, the question on retirement was unavailable, so labour force status was recoded into currently working and currently not working.

| **Supplementary Table S4 Univariate analysis of covariates and cognitive impairment** | | | | | |
| --- | --- | --- | --- | --- | --- |
| **Variables** | **CHARLS** | **ELSA** | **HRS** | **MHAS** | **SHARE** |
|  | **OR/β (95% CI) P value** | **OR/β (95% CI) P value** | **OR/β (95% CI) P value** | **OR/β (95% CI) P value** | **OR/β (95% CI) P value** |
| **Age** | 1.00 (0.99, 1.01) 0.777 | 1.02 (1.01, 1.03) <0.001 | 1.02 (1.01, 1.02) <0.001 | 1.01 (1.00, 1.01) 0.019 | 1.01 (1.00, 1.01) <0.001 |
| **Gender** |  |  |  |  |  |
| **Male** | Ref | Ref | Ref | Ref | Ref |
| **Female** | 2.06 (1.89, 2.24) <0.001 | 0.76 (0.69, 0.85) <0.001 | 0.94 (0.88, 1.01) 0.107 | 0.89 (0.82, 0.97) 0.010 | 1.09 (1.02, 1.17) 0.018 |
| **Education** |  |  |  |  |  |
| **Less than upper secondary** | Ref | Ref | Ref | Ref | Ref |
| **Upper secondary and vocational training** | 0.18 (0.13, 0.24) <0.001 | 0.45 (0.40, 0.51) <0.001 | 0.29 (0.27, 0.32) <0.001 | 0.22 (0.14, 0.35) <0.001 | 0.39 (0.36, 0.42) <0.001 |
| **Tertiary** | 0.10 (0.06, 0.18) <0.001 | 0.22 (0.18, 0.26) <0.001 | 0.14 (0.13, 0.15) <0.001 | 0.10 (0.07, 0.15) <0.001 | 0.17 (0.15, 0.19) <0.001 |
| **Labour force status** |  |  |  |  |  |
| **Currently not working** | Ref | Ref | Ref | Ref | Ref |
| **Currently working without retirement** | 1.08 (0.97, 1.19) 0.154 | 0.71 (0.62, 0.81) <0.001 | 0.56 (0.50, 0.63) <0.001 | 0.95 (0.87, 1.04) 0.262 | 0.62 (0.55, 0.71) <0.001 |
| **Currently working after retirement** | 0.35 (0.26, 0.47) <0.001 | 0.62 (0.46, 0.82) <0.001 | 0.57 (0.52, 0.63) <0.001 | —* | 0.44 (0.36, 0.55) <0.001 |
| **Household wealth** |  |  |  |  |  |
| **Low tertile** | Ref | Ref | Ref | Ref | Ref |
| **Medium tertile** | 0.78 (0.70, 0.87) <0.001 | 0.64 (0.57, 0.71) <0.001 | 0.50 (0.47, 0.53) <0.001 | 0.89 (0.81, 0.99) 0.038 | 0.65 (0.59, 0.70) <0.001 |
| **High tertile** | 0.49 (0.43, 0.55) <0.001 | 0.40 (0.35, 0.45) <0.001 | 0.29 (0.26, 0.31) <0.001 | 0.65 (0.58, 0.72) <0.001 | 0.35 (0.32, 0.39) <0.001 |
| **Married or partnered** |  |  |  |  |  |
| **No** | Ref | Ref | Ref | Ref | Ref |
| **Yes** | 0.70 (0.63, 0.78) <0.001 | 0.85 (0.76, 0.95) 0.004 | 0.64 (0.60, 0.68) <0.001 | 0.94 (0.86, 1.03) 0.199 | 0.79 (0.72, 0.85) <0.001 |
| **Co-residence with children** |  |  |  |  |  |
| **No** | Ref | Ref | Ref | Ref | Ref |
| **Yes** | 1.07 (0.98, 1.17) 0.147 | 1.20 (0.73, 1.98) 0.474 | 0.89 (0.83, 0.95) <0.001 | 1.08 (0.98, 1.19) 0.104 | 1.49 (1.36, 1.62) <0.001 |
| **Smoking** |  |  |  |  |  |
| **No** | Ref | Ref | Ref | Ref | Ref |
| **Yes** | 0.81 (0.72, 0.90) <0.001 | 1.72 (1.46, 2.02) <0.001 | 1.52 (1.37, 1.69) <0.001 | 0.89 (0.77, 1.03) 0.129 | 0.96 (0.87, 1.06) 0.432 |
| **Drinking** |  |  |  |  |  |
| **No** | Ref | Ref | Ref | Ref | Ref |
| **Yes** | 0.65 (0.59, 0.72) <0.001 | 0.53 (0.47, 0.60) <0.001 | 0.54 (0.51, 0.58) <0.001 | 0.86 (0.78, 0.95) 0.002 | 0.55 (0.51, 0.59) <0.001 |
| **Hypertension** |  |  |  |  |  |
| **No** | Ref | Ref | Ref | Ref | Ref |
| **Yes** | 1.00 (0.92, 1.09) 0.962 | 1.24 (1.12, 1.37) <0.001 | 1.40 (1.30, 1.51) <0.001 | 1.08 (0.98, 1.18) 0.120 | 1.12 (1.05, 1.21) 0.001 |
| **Stroke** |  |  |  |  |  |
| **No** | Ref | Ref | Ref | Ref | Ref |
| **Yes** | 1.17 (0.98, 1.39) 0.082 | 1.88 (1.54, 2.29) <0.001 | 1.99 (1.82, 2.17) <0.001 | 1.56 (1.30, 1.88) <0.001 | 1.92 (1.69, 2.18) <0.001 |
| **Cancer** |  |  |  |  |  |
| **No** | Ref | Ref | Ref | Ref | Ref |
| **Yes** | 0.95 (0.69, 1.30) 0.736 | 0.98 (0.85, 1.13) 0.755 | 0.88 (0.81, 0.95) 0.001 | 0.70 (0.57, 0.87) 0.002 | 0.82 (0.73, 0.93) 0.003 |
| **Depressive symptom** |  |  |  |  |  |
| **No** | Ref | Ref | Ref | Ref | Ref |
| **Yes** | 1.79 (1.64, 1.96) <0.001 | 1.80 (1.61, 2.01) <0.001 | 1.65 (1.55, 1.76) <0.001 | 1.52 (1.40, 1.66) <0.001 | 2.39 (2.22, 2.57) <0.001 |

Notes: CHARLS: China Health and Retirement Longitudinal Study; ELSA: English Longitudinal Study of Ageing; HRS: Health and Retirement Study; MHAS: Mexican Health and Aging Study; SHARE: Survey of Health, Ageing and Retirement in Europe.

*For MHAS, the question on retirement was unavailable, so labour force status was recoded into currently working and currently not working.

| **Supplementary Table S5 Univariate analysis of covariates and oriention scores** | | | | | |
| --- | --- | --- | --- | --- | --- |
| **Variables** | **CHARLS** | **ELSA** | **HRS** | **MHAS** | **SHARE** |
|  | **OR/β (95% CI) P value** | **OR/β (95% CI) P value** | **OR/β (95% CI) P value** | **OR/β (95% CI) P value** | **OR/β (95% CI) P value** |
| **Age** | -0.02 (-0.02, -0.01) <0.001 | -0.01 (-0.01, -0.01) <0.001 | -0.02 (-0.02, -0.02) <0.001 | -0.03 (-0.03, -0.03) <0.001 | -0.01 (-0.01, -0.01) <0.001 |
| **Gender** |  |  |  |  |  |
| **Male** | Ref | Ref | Ref | Ref | Ref |
| **Female** | -0.30 (-0.34, -0.25) <0.001 | 0.06 (0.04, 0.07) <0.001 | 0.06 (0.04, 0.08) <0.001 | -0.07 (-0.10, -0.04) <0.001 | -0.00 (-0.02, 0.01) 0.658 |
| **Education** |  |  |  |  |  |
| **Less than upper secondary** | Ref | Ref | Ref | Ref | Ref |
| **Upper secondary and vocational training** | 0.54 (0.48, 0.60) <0.001 | 0.07 (0.05, 0.09) <0.001 | 0.21 (0.18, 0.24) <0.001 | 0.36 (0.30, 0.42) <0.001 | 0.07 (0.06, 0.09) <0.001 |
| **Tertiary** | 0.61 (0.51, 0.72) <0.001 | 0.12 (0.09, 0.14) <0.001 | 0.27 (0.24, 0.30) <0.001 | 0.43 (0.40, 0.46) <0.001 | 0.10 (0.09, 0.12) <0.001 |
| **Labour force status** |  |  |  |  |  |
| **Currently not working** | Ref | Ref | Ref | Ref | Ref |
| **Currently working without retirement** | -0.12 (-0.17, -0.08) <0.001 | 0.08 (0.06, 0.10) <0.001 | 0.22 (0.20, 0.24) <0.001 | 0.14 (0.12, 0.17) <0.001 | 0.11 (0.10, 0.13) <0.001 |
| **Currently working after retirement** | 0.34 (0.25, 0.43) <0.001 | 0.04 (-0.00, 0.08) 0.083 | 0.17 (0.15, 0.19) <0.001 | —* | 0.08 (0.06, 0.10) <0.001 |
| **Household wealth** |  |  |  |  |  |
| **Low tertile** | Ref | Ref | Ref | Ref | Ref |
| **Medium tertile** | 0.10 (0.04, 0.16) <0.001 | 0.03 (0.01, 0.05) 0.005 | 0.08 (0.06, 0.10) <0.001 | 0.10 (0.06, 0.14) <0.001 | 0.02 (0.00, 0.03) 0.040 |
| **High tertile** | 0.31 (0.25, 0.36) <0.001 | 0.06 (0.04, 0.08) <0.001 | 0.13 (0.11, 0.15) <0.001 | 0.20 (0.16, 0.23) <0.001 | 0.03 (0.01, 0.04) 0.001 |
| **Married or partnered** |  |  |  |  |  |
| **No** | Ref | Ref | Ref | Ref | Ref |
| **Yes** | 0.18 (0.13, 0.23) <0.001 | 0.03 (0.01, 0.05) 0.010 | 0.10 (0.08, 0.12) <0.001 | 0.18 (0.15, 0.21) <0.001 | 0.09 (0.07, 0.11) <0.001 |
| **Co-residence with children** |  |  |  |  |  |
| **No** | Ref | Ref | Ref | Ref | Ref |
| **Yes** | -0.07 (-0.12, -0.03) 0.001 | 0.03 (-0.04, 0.09) 0.4355 | 0.02 (0.00, 0.04) 0.049 | 0.01 (-0.03, 0.04) 0.683 | -0.00 (-0.02, 0.02) 0.814 |
| **Smoking** |  |  |  |  |  |
| **No** | Ref | Ref | Ref | Ref | Ref |
| **Yes** | 0.05 (0.00, 0.10) 0.043 | -0.03 (-0.06, 0.00) 0.060 | -0.01 (-0.05, 0.02) 0.433 | 0.10 (0.05, 0.14) <0.001 | 0.02 (0.01, 0.04) 0.006 |
| **Drinking** |  |  |  |  |  |
| **No** | Ref | Ref | Ref | Ref | Ref |
| **Yes** | 0.13 (0.09, 0.18) <0.001 | 0.06 (0.03, 0.09) <0.001 | 0.12 (0.10, 0.14) <0.001 | 0.15 (0.12, 0.18) <0.001 | 0.05 (0.03, 0.06) <0.001 |
| **Hypertension** |  |  |  |  |  |
| **No** | Ref | Ref | Ref | Ref | Ref |
| **Yes** | 0.03 (-0.01, 0.07) 0.1100 | -0.03 (-0.04, -0.01) 0.004 | -0.05 (-0.07, -0.03) <0.001 | -0.07 (-0.10, -0.04) <0.001 | -0.02 (-0.04, -0.01) <0.001 |
| **Stroke** |  |  |  |  |  |
| **No** | Ref | Ref | Ref | Ref | Ref |
| **Yes** | -0.05 (-0.13, 0.03) 0.253 | -0.11 (-0.16, -0.06) <0.001 | -0.27 (-0.31, -0.23) <0.001 | -0.32 (-0.41, -0.24) <0.001 | -0.17 (-0.21, -0.13) <0.001 |
| **Cancer** |  |  |  |  |  |
| **No** | Ref | Ref | Ref | Ref | Ref |
| **Yes** | 0.12 (-0.02, 0.26) 0.087 | -0.04 (-0.07, -0.01) 0.006 | -0.03 (-0.05, -0.01) 0.016 | 0.06 (-0.00, 0.13) 0.0578 | -0.01 (-0.03, 0.01) 0.394 |
| **Depressive symptom** |  |  |  |  |  |
| **No** | Ref | Ref | Ref | Ref | Ref |
| **Yes** | -0.29 (-0.34, -0.25) <0.001 | -0.06 (-0.08, -0.03) <0.001 | -0.13 (-0.15, -0.10) <0.001 | -0.19 (-0.22, -0.16) <0.001 | -0.13 (-0.15, -0.12) <0.001 |

Notes: CHARLS: China Health and Retirement Longitudinal Study; ELSA: English Longitudinal Study of Ageing; HRS: Health and Retirement Study; MHAS: Mexican Health and Aging Study; SHARE: Survey of Health, Ageing and Retirement in Europe.

*For MHAS, the question on retirement was unavailable, so labour force status was recoded into currently working and currently not working.

| **Supplementary Table S6 Univariate analysis of covariates and memory scores** | | | | | |
| --- | --- | --- | --- | --- | --- |
| **Variables** | **CHARLS** | **ELSA** | **HRS** | **MHAS** | **SHARE** |
|  | **OR/β (95% CI) P value** | **OR/β (95% CI) P value** | **OR/β (95% CI) P value** | **OR/β (95% CI) P value** | **OR/β (95% CI) P value** |
| **Age** | -0.13 (-0.14, -0.12) <0.001 | -0.18 (-0.19, -0.17) <0.001 | -0.17 (-0.18, -0.17) <0.001 | -0.16 (-0.16, -0.15) <0.001 | -0.17 (-0.18, -0.16) <0.001 |
| **Gender** |  |  |  |  |  |
| **Male** | Ref | Ref | Ref | Ref | Ref |
| **Female** | -0.15 (-0.27, -0.03) 0.017 | 1.03 (0.88, 1.18) <0.001 | 0.90 (0.80, 1.00) <0.001 | 0.95 (0.84, 1.06) <0.001 | 0.38 (0.29, 0.47) <0.001 |
| **Education** |  |  |  |  |  |
| **Less than upper secondary** | Ref | Ref | Ref | Ref | Ref |
| **Upper secondary and vocational training** | 2.19 (1.92, 2.47) <0.001 | 1.91 (1.74, 2.08) <0.001 | 1.69 (1.57, 1.82) <0.001 | 1.84 (1.55, 2.14) <0.001 | 1.64 (1.55, 1.74) <0.001 |
| **Tertiary** | 2.67 (2.09, 3.25) <0.001 | 3.11 (2.90, 3.32) <0.001 | 2.85 (2.72, 2.97) <0.001 | 2.30 (2.14, 2.46) <0.001 | 2.84 (2.73, 2.95) <0.001 |
| **Labour force status** |  |  |  |  |  |
| **Currently not working** | Ref | Ref | Ref | Ref | Ref |
| **Currently working without retirement** | 0.07 (-0.09, 0.23) 0.395 | 1.13 (0.99, 1.27) <0.001 | 1.44 (1.31, 1.57) <0.001 | 0.40 (0.30, 0.50) <0.001 | 1.86 (1.72, 1.99) <0.001 |
| **Currently working after retirement** | 1.22 (0.94, 1.49) <0.001 | 0.91 (0.68, 1.15) <0.001 | 1.08 (0.97, 1.19) <0.001 | —* | 1.57 (1.38, 1.77) <0.001 |
| **Household wealth** |  |  |  |  |  |
| **Low tertile** | Ref | Ref | Ref | Ref | Ref |
| **Medium tertile** | 0.31 (0.16, 0.46) <0.001 | 0.47 (0.34, 0.60) <0.001 | 0.60 (0.52, 0.68) <0.001 | 0.09 (-0.04, 0.23) 0.1687 | 0.45 (0.34, 0.56) <0.001 |
| **High tertile** | 1.14 (0.96, 1.32) <0.001 | 1.13 (0.98, 1.27) <0.001 | 1.10 (1.00, 1.20) <0.001 | 0.62 (0.48, 0.75) <0.001 | 1.33 (1.22, 1.44) <0.001 |
| **Married or partnered** |  |  |  |  |  |
| **No** | Ref | Ref | Ref | Ref | Ref |
| **Yes** | 0.96 (0.80, 1.13) <0.001 | 0.82 (0.66, 0.97) <0.001 | 0.87 (0.78, 0.96) <0.001 | 0.40 (0.29, 0.51) <0.001 | 0.86 (0.75, 0.97) <0.001 |
| **Co-residence with children** |  |  |  |  |  |
| **No** | Ref | Ref | Ref | Ref | Ref |
| **Yes** | -0.07 (-0.19, 0.06) 0.316 | 0.52 (-0.16, 1.19) 0.132 | 0.48 (0.39, 0.57) <0.001 | 0.20 (0.08, 0.31) 0.001 | -0.32 (-0.44, -0.20) <0.001 |
| **Smoking** |  |  |  |  |  |
| **No** | Ref | Ref | Ref | Ref | Ref |
| **Yes** | -0.19 (-0.35, -0.03) 0.017 | -0.47 (-0.71, -0.23) <0.001 | -0.11 (-0.25, 0.04) 0.156 | 0.21 (0.05, 0.38) 0.012 | 0.52 (0.40, 0.64) <0.001 |
| **Drinking** |  |  |  |  |  |
| **No** | Ref | Ref | Ref | Ref | Ref |
| **Yes** | 0.36 (0.22, 0.49) <0.001 | 1.17 (0.98, 1.36) <0.001 | 0.91 (0.83, 0.99) <0.001 | 0.34 (0.24, 0.44) <0.001 | 1.04 (0.95, 1.13) <0.001 |
| **Hypertension** |  |  |  |  |  |
| **No** | Ref | Ref | Ref | Ref | Ref |
| **Yes** | -0.16 (-0.29, -0.02) 0.020 | -0.70 (-0.84, -0.56) <0.001 | -0.66 (-0.76, -0.56) <0.001 | -0.40 (-0.51, -0.29) <0.001 | -0.56 (-0.64, -0.47) <0.001 |
| **Stroke** |  |  |  |  |  |
| **No** | Ref | Ref | Ref | Ref | Ref |
| **Yes** | -0.45 (-0.72, -0.18) 0.001 | -1.79 (-2.11, -1.47) <0.001 | -1.44 (-1.58, -1.30) <0.001 | -0.97 (-1.22, -0.71) <0.001 | -1.46 (-1.67, -1.26) <0.001 |
| **Cancer** |  |  |  |  |  |
| **No** | Ref | Ref | Ref | Ref | Ref |
| **Yes** | 0.07 (-0.37, 0.52) 0.749 | -0.49 (-0.68, -0.30) <0.001 | -0.33 (-0.44, -0.22) <0.001 | 0.36 (0.11, 0.61) 0.004 | 0.06 (-0.09, 0.21) 0.467 |
| **Depressive symptom** |  |  |  |  |  |
| **No** | Ref | Ref | Ref | Ref | Ref |
| **Yes** | -0.99 (-1.12, -0.87) <0.001 | -0.77 (-0.91, -0.63) <0.001 | -0.72 (-0.80, -0.64) <0.001 | -0.59 (-0.68, -0.49) <0.001 | -1.42 (-1.52, -1.32) <0.001 |

Notes: CHARLS: China Health and Retirement Longitudinal Study; ELSA: English Longitudinal Study of Ageing; HRS: Health and Retirement Study; MHAS: Mexican Health and Aging Study; SHARE: Survey of Health, Ageing and Retirement in Europe.

*For MHAS, the question on retirement was unavailable, so labour force status was recoded into currently working and currently not working.

| **Supplementary Table S7 Univariate analysis of covariates and executive scores** | | | | | |
| --- | --- | --- | --- | --- | --- |
| **Variables** | **CHARLS** | **ELSA** | **HRS** | **MHAS** | **SHARE** |
|  | **OR/β (95% CI) P value** | **OR/β (95% CI) P value** | **OR/β (95% CI) P value** | **OR/β (95% CI) P value** | **OR/β (95% CI) P value** |
| **Age** | -0.04 (-0.04, -0.03) <0.001 | -0.01 (-0.02, -0.01) <0.001 | -0.03 (-0.03, -0.03) <0.001 | -0.04 (-0.04, -0.04) <0.001 | -0.03 (-0.04, -0.03) <0.001 |
| **Gender** |  |  |  |  |  |
| **Male** | Ref | Ref | Ref | Ref | Ref |
| **Female** | -0.76 (-0.83, -0.68) <0.001 | -0.22 (-0.26, -0.18) <0.001 | -0.46 (-0.52, -0.41) <0.001 | -0.46 (-0.51, -0.40) <0.001 | -0.30 (-0.34, -0.27) <0.001 |
| **Education** |  |  |  |  |  |
| **Less than upper secondary** | Ref | Ref | Ref | Ref | Ref |
| **Upper secondary and vocational training** | 0.78 (0.68, 0.88) <0.001 | 0.36 (0.31, 0.41) <0.001 | 1.21 (1.14, 1.28) <0.001 | 0.98 (0.83, 1.12) <0.001 | 0.65 (0.61, 0.68) <0.001 |
| **Tertiary** | 0.95 (0.74, 1.15) <0.001 | 0.59 (0.53, 0.64) <0.001 | 1.79 (1.73, 1.86) <0.001 | 1.28 (1.20, 1.35) <0.001 | 0.84 (0.80, 0.88) <0.001 |
| **Labour force status** |  |  |  |  |  |
| **Currently not working** | Ref | Ref | Ref | Ref | Ref |
| **Currently working without retirement** | 0.09 (0.02, 0.17) 0.014 | 0.08 (0.04, 0.13) <0.001 | 0.36 (0.30, 0.42) <0.001 | 0.24 (0.19, 0.30) <0.001 | 0.39 (0.35, 0.44) <0.001 |
| **Currently working after retirement** | 0.56 (0.44, 0.68) <0.001 | 0.11 (0.03, 0.20) 0.007 | 0.33 (0.28, 0.38) <0.001 | —* | 0.43 (0.38, 0.49) <0.001 |
| **Household wealth** |  |  |  |  |  |
| **Low tertile** | Ref | Ref | Ref | Ref | Ref |
| **Medium tertile** | 0.14 (0.06, 0.22) <0.001 | 0.19 (0.15, 0.23) <0.001 | 0.40 (0.36, 0.44) <0.001 | 0.06 (-0.01, 0.13) 0.103 | 0.23 (0.19, 0.27) <0.001 |
| **High tertile** | 0.43 (0.35, 0.50) <0.001 | 0.30 (0.26, 0.34) <0.001 | 0.69 (0.64, 0.73) <0.001 | 0.39 (0.33, 0.46) <0.001 | 0.48 (0.44, 0.52) <0.001 |
| **Married or partnered** |  |  |  |  |  |
| **No** | Ref | Ref | Ref | Ref | Ref |
| **Yes** | 0.51 (0.43, 0.59) <0.001 | 0.10 (0.06, 0.15) <0.001 | 0.43 (0.38, 0.48) <0.001 | 0.38 (0.32, 0.43) <0.001 | 0.31 (0.26, 0.35) <0.001 |
| **Co-residence with children** |  |  |  |  |  |
| **No** | Ref | Ref | Ref | Ref | Ref |
| **Yes** | -0.02 (-0.08, 0.04) 0.466 | -0.20 (-0.41, 0.02) 0.072 | 0.16 (0.12, 0.21) <0.001 | 0.02 (-0.04, 0.08) 0.598 | -0.16 (-0.21, -0.12) <0.001 |
| **Smoking** |  |  |  |  |  |
| **No** | Ref | Ref | Ref | Ref | Ref |
| **Yes** | 0.33 (0.26, 0.40) <0.001 | -0.05 (-0.12, 0.01) 0.111 | -0.16 (-0.23, -0.08) <0.001 | 0.26 (0.17, 0.34) <0.001 | 0.19 (0.15, 0.23) <0.001 |
| **Drinking** |  |  |  |  |  |
| **No** | Ref | Ref | Ref | Ref | Ref |
| **Yes** | 0.42 (0.35, 0.49) <0.001 | 0.29 (0.23, 0.35) <0.001 | 0.41 (0.37, 0.45) <0.001 | 0.27 (0.22, 0.33) <0.001 | 0.37 (0.33, 0.40) <0.001 |
| **Hypertension** |  |  |  |  |  |
| **No** | Ref | Ref | Ref | Ref | Ref |
| **Yes** | -0.02 (-0.08, 0.04) 0.598 | -0.07 (-0.11, -0.03) <0.001 | -0.31 (-0.36, -0.27) <0.001 | -0.22 (-0.28, -0.17) <0.001 | -0.16 (-0.20, -0.13) <0.001 |
| **Stroke** |  |  |  |  |  |
| **No** | Ref | Ref | Ref | Ref | Ref |
| **Yes** | -0.11 (-0.24, 0.02) 0.110 | -0.18 (-0.28, -0.08) <0.001 | -0.46 (-0.53, -0.39) <0.001 | -0.29 (-0.41, -0.16) <0.001 | -0.35 (-0.44, -0.27) <0.001 |
| **Cancer** |  |  |  |  |  |
| **No** | Ref | Ref | Ref | Ref | Ref |
| **Yes** | 0.04 (-0.17, 0.26) 0.679 | -0.01 (-0.06, 0.04) 0.720 | 0.11 (0.06, 0.17) <0.001 | 0.07 (-0.05, 0.20) 0.241 | 0.11 (0.06, 0.16) <0.001 |
| **Depressive symptom** |  |  |  |  |  |
| **No** | Ref | Ref | Ref | Ref | Ref |
| **Yes** | -0.43 (-0.49, -0.38) <0.001 | -0.17 (-0.21, -0.12) <0.001 | -0.31 (-0.35, -0.27) <0.001 | -0.31 (-0.36, -0.26) <0.001 | -0.55 (-0.60, -0.51) <0.001 |

Notes: CHARLS: China Health and Retirement Longitudinal Study; ELSA: English Longitudinal Study of Ageing; HRS: Health and Retirement Study; MHAS: Mexican Health and Aging Study; SHARE: Survey of Health, Ageing and Retirement in Europe.

*For MHAS, the question on retirement was unavailable, so labour force status was recoded into currently working and currently not working.

| **Supplementary Table S8 Univariate analysis of covariates and total cognitive scores** | | | | | |
| --- | --- | --- | --- | --- | --- |
| **Variables** | **CHARLS** | **ELSA** | **HRS** | **MHAS** | **SHARE** |
|  | **OR/β (95% CI) P value** | **OR/β (95% CI) P value** | **OR/β (95% CI) P value** | **OR/β (95% CI) P value** | **OR/β (95% CI) P value** |
| **Age** | -0.18 (-0.20, -0.17) <0.001 | -0.20 (-0.21, -0.19) <0.001 | -0.23 (-0.24, -0.22) <0.001 | -0.22 (-0.23, -0.22) <0.001 | -0.22 (-0.22, -0.21) <0.001 |
| **Gender** |  |  |  |  |  |
| **Male** | Ref | Ref | Ref | Ref | Ref |
| **Female** | -1.21 (-1.41, -1.00) <0.001 | 0.86 (0.69, 1.03) <0.001 | 0.50 (0.35, 0.65) <0.001 | 0.43 (0.27, 0.59) <0.001 | 0.08 (-0.03, 0.19) 0.171 |
| **Education** |  |  |  |  |  |
| **Less than upper secondary** | Ref | Ref | Ref | Ref | Ref |
| **Upper secondary and vocational training** | 3.49 (3.14, 3.84) <0.001 | 2.36 (2.16, 2.56) <0.001 | 3.11 (2.93, 3.30) <0.001 | 3.19 (2.82, 3.56) <0.001 | 2.36 (2.24, 2.48) <0.001 |
| **Tertiary** | 4.20 (3.47, 4.93) <0.001 | 3.83 (3.60, 4.07) <0.001 | 4.92 (4.75, 5.10) <0.001 | 4.02 (3.82, 4.22) <0.001 | 3.78 (3.65, 3.91) <0.001 |
| **Labour force status** |  |  |  |  |  |
| **Currently not working** | Ref | Ref | Ref | Ref | Ref |
| **Currently working without retirement** | 0.07 (-0.17, 0.31) 0.577 | 1.27 (1.11, 1.43) <0.001 | 1.87 (1.72, 2.03) <0.001 | 0.71 (0.58, 0.84) <0.001 | 2.37 (2.21, 2.52) <0.001 |
| **Currently working after retirement** | 2.13 (1.72, 2.53) <0.001 | 0.99 (0.72, 1.26) <0.001 | 1.46 (1.33, 1.59) <0.001 | —* | 2.09 (1.87, 2.30) <0.001 |
| **Household wealth** |  |  |  |  |  |
| **Low tertile** | Ref | Ref | Ref | Ref | Ref |
| **Medium tertile** | 0.55 (0.33, 0.77) <0.001 | 0.67 (0.52, 0.81) <0.001 | 1.01 (0.90, 1.12) <0.001 | 0.25 (0.06, 0.44) 0.010 | 0.69 (0.56, 0.83) <0.001 |
| **High tertile** | 1.86 (1.61, 2.11) <0.001 | 1.45 (1.28, 1.61) <0.001 | 1.81 (1.68, 1.94) <0.001 | 1.21 (1.03, 1.40) <0.001 | 1.83 (1.70, 1.96) <0.001 |
| **Married or partnered** |  |  |  |  |  |
| **No** | Ref | Ref | Ref | Ref | Ref |
| **Yes** | 1.66 (1.42, 1.89) <0.001 | 0.96 (0.78, 1.13) <0.001 | 1.41 (1.29, 1.54) <0.001 | 0.94 (0.79, 1.09) <0.001 | 1.26 (1.12, 1.40) <0.001 |
| **Co-residence with children** |  |  |  |  |  |
| **No** | Ref | Ref | Ref | Ref | Ref |
| **Yes** | -0.15 (-0.34, 0.04) 0.128 | 0.33 (-0.45, 1.12) 0.406 | 0.65 (0.53, 0.77) <0.001 | 0.23 (0.06, 0.40) 0.008 | -0.49 (-0.64, -0.34) <0.001 |
| **Smoking** |  |  |  |  |  |
| **No** | Ref | Ref | Ref | Ref | Ref |
| **Yes** | 0.20 (-0.03, 0.42) 0.093 | -0.52 (-0.79, -0.25) <0.001 | -0.24 (-0.44, -0.04) 0.021 | 0.54 (0.31, 0.77) <0.001 | 0.73 (0.59, 0.88) <0.001 |
| **Drinking** |  |  |  |  |  |
| **No** | Ref | Ref | Ref | Ref | Ref |
| **Yes** | 0.91 (0.70, 1.12) <0.001 | 1.51 (1.28, 1.73) <0.001 | 1.37 (1.26, 1.48) <0.001 | 0.65 (0.52, 0.79) <0.001 | 1.46 (1.35, 1.57) <0.001 |
| **Hypertension** |  |  |  |  |  |
| **No** | Ref | Ref | Ref | Ref | Ref |
| **Yes** | -0.15 (-0.33, 0.03) 0.103 | -0.81 (-0.97, -0.65) <0.001 | -1.04 (-1.17, -0.91) <0.001 | -0.69 (-0.84, -0.53) <0.001 | -0.74 (-0.85, -0.63) <0.001 |
| **Stroke** |  |  |  |  |  |
| **No** | Ref | Ref | Ref | Ref | Ref |
| **Yes** | -0.62 (-1.01, -0.22) 0.002 | -2.11 (-2.48, -1.74) <0.001 | -2.16 (-2.36, -1.96) <0.001 | -1.55 (-1.90, -1.19) <0.001 | -1.99 (-2.26, -1.72) <0.001 |
| **Cancer** |  |  |  |  |  |
| **No** | Ref | Ref | Ref | Ref | Ref |
| **Yes** | 0.23 (-0.41, 0.87) 0.489 | -0.54 (-0.76, -0.32) <0.001 | -0.30 (-0.45, -0.15) <0.001 | 0.46 (0.12, 0.79) 0.007 | 0.16 (-0.03, 0.34) 0.092 |
| **Depressive symptom** |  |  |  |  |  |
| **No** | Ref | Ref | Ref | Ref | Ref |
| **Yes** | -1.71 (-1.89, -1.52) <0.001 | -0.94 (-1.09, -0.78) <0.001 | -1.04 (-1.15, -0.94) <0.001 | -0.95 (-1.07, -0.82) <0.001 | -2.11 (-2.24, -1.98) <0.001 |

Notes: CHARLS: China Health and Retirement Longitudinal Study; ELSA: English Longitudinal Study of Ageing; HRS: Health and Retirement Study; MHAS: Mexican Health and Aging Study; SHARE: Survey of Health, Ageing and Retirement in Europe.

*For MHAS, the question on retirement was unavailable, so labour force status was recoded into currently working and currently not working.

| **Supplementary Table S9 Association between** **digital exclusion and oriention scores by age, gender, education, labour force status, household wealth, married or partnered, co-residence with children, smoking, drinking, hypertension, stroke, cancer and depressive symptoms** | | | | | | | | | | |
| --- | --- | --- | --- | --- | --- | --- | --- | --- | --- | --- |
| **Subgroups** | **CHARLS** | | **ELSA** | | **HRS** | | **MHAS** | | **SHARE** | |
|  | **OR (95% CI)** | ***P* for interaction** | **OR (95% CI)** | ***P* for interaction** | **OR (95% CI)** | ***P* for interaction** | **OR (95% CI)** | ***P* for interaction** | **OR (95% CI)** | ***P* for interaction** |
| **Age** |  | 0.245 |  | <0.001 |  | <0.001 |  | 0.298 |  | <0.001 |
| **60-79** | -0.22 (-0.29, -0.14) |  | -0.06 (-0.09, -0.03) |  | -0.09 (-0.11, -0.07) |  | -0.20 (-0.23, -0.17) |  | -0.04 (-0.05, -0.03) |  |
| **≥80** | -0.74 (-1.33, -0.16) |  | -0.15 (-0.21, -0.09) |  | -0.28 (-0.32, -0.23) |  | -0.23 (-0.33, -0.13) |  | -0.19 (-0.25, -0.13) |  |
| **Gender** |  | 0.572 |  | 0.174 |  | 0.016 |  | 0.005 |  | 0.767 |
| **Male** | -0.17 (-0.26, -0.08) |  | -0.10 (-0.14, -0.05) |  | -0.14 (-0.16, -0.11) |  | -0.16 (-0.20, -0.12) |  | -0.03 (-0.05, -0.01) |  |
| **Female** | -0.21 (-0.33, -0.10) |  | -0.06 (-0.09, -0.03) |  | -0.09 (-0.11, -0.06) |  | -0.22 (-0.26, -0.19) |  | -0.04 (-0.05, -0.02) |  |
| **Education** |  | <0.001 |  | 0.013 |  | <0.001 |  | 0.117 |  | 0.515 |
| **Less than upper secondary** | -0.30 (-0.40, -0.21) |  | -0.10 (-0.14, -0.06) |  | -0.04 (-0.10, 0.02) |  | -0.21 (-0.23, -0.18) |  | -0.03 (-0.05, -0.01) |  |
| **Upper secondary and vocational training** | -0.07 (-0.18, 0.04) |  | -0.06 (-0.09, -0.02) |  | -0.10 (-0.12, -0.07) |  | -0.11 (-0.23, 0.02) |  | -0.04 (-0.06, -0.02) |  |
| **Tertiary** | 0.23 (0.00, 0.45) |  | -0.16 (-0.29, -0.03) |  | -0.15 (-0.18, -0.13) |  | -0.13 (-0.22, -0.05) |  | -0.05 (-0.08, -0.02) |  |
| **Labour force status** |  | 0.128 |  | 0.876 |  | 0.008 |  | 0.209 |  | 0.458 |
| **Currently not working** | -0.14 (-0.21, -0.06) |  | -0.08 (-0.11, -0.05) |  | -0.12 (-0.14, -0.10) |  | -0.21 (-0.24, -0.17) |  | -0.04 (-0.05, -0.02) |  |
| **Currently working without retirement** | -0.34 (-0.52, -0.16) |  | -0.06 (-0.13, 0.02) |  | -0.03 (-0.07, 0.01) |  | -0.18 (-0.23, -0.14) |  | -0.00 (-0.03, 0.03) |  |
| **Currently working after retirement** | -0.11 (-0.28, 0.06) |  | -0.08 (-0.24, 0.09) |  | -0.08 (-0.12, -0.04) |  | —* |  | -0.04 (-0.09, 0.02) |  |
| **Household wealth** |  | 0.072 |  | 0.309 |  | 0.026 |  | <0.001 |  | <0.001 |
| **Low tertile** | -0.23 (-0.39, -0.07) |  | -0.09 (-0.13, -0.05) |  | -0.07 (-0.10, -0.04) |  | -0.25 (-0.30, -0.20) |  | 0.00 (-0.02, 0.03) |  |
| **Medium tertile** | -0.35 (-0.48, -0.22) |  | -0.06 (-0.10, -0.01) |  | -0.11 (-0.14, -0.08) |  | -0.24 (-0.28, -0.19) |  | -0.04 (-0.06, -0.02) |  |
| **High tertile** | -0.13 (-0.21, -0.04) |  | -0.09 (-0.14, -0.03) |  | -0.14 (-0.17, -0.11) |  | -0.12 (-0.17, -0.08) |  | -0.07 (-0.09, -0.05) |  |
| **Married or partnered** |  | 0.475 |  | 0.526 |  | 0.142 |  | 0.611 |  | 0.226 |
| **No** | -0.10 (-0.34, 0.15) |  | -0.07 (-0.11, -0.03) |  | -0.10 (-0.13, -0.07) |  | -0.20 (-0.25, -0.16) |  | -0.06 (-0.09, -0.02) |  |
| **Yes** | -0.20 (-0.28, -0.13) |  | -0.09 (-0.12, -0.05) |  | —# |  | -0.20 (-0.23, -0.17) |  | -0.03 (-0.04, -0.02) |  |
| **Co-residence with children** |  | 0.326 |  | 0.403 |  | 0.555 |  | 0.507 |  | 0.127 |
| **No** | -0.15 (-0.24, -0.06) |  | -0.08 (-0.11, -0.05) |  | —# |  | -0.22 (-0.27, -0.17) |  | -0.04 (-0.05, -0.02) |  |
| **Yes** | -0.28 (-0.42, -0.15) |  | 0.10 (-0.12, 0.33) |  | -0.11 (-0.13, -0.09) |  | -0.19 (-0.22, -0.16) |  | -0.01 (-0.04, 0.03) |  |
| **Smoking** |  | 0.976 |  | 0.080 |  | 0.002 |  | 0.598 |  | 0.253 |
| **No** | -0.19 (-0.28, -0.10) |  | -0.09 (-0.11, -0.06) |  | -0.12 (-0.13, -0.10) |  | -0.20 (-0.23, -0.17) |  | -0.04 (-0.05, -0.02) |  |
| **Yes** | -0.19 (-0.33, -0.06) |  | -0.03 (-0.11, 0.04) |  | -0.04 (-0.10, 0.01) |  | -0.18 (-0.26, -0.10) |  | -0.01 (-0.04, 0.02) |  |
| **Drinking** |  | 0.456 |  | 0.138 |  | 0.918 |  | 0.917 |  | 0.159 |
| **No** | -0.15 (-0.25, -0.04) |  | -0.12 (-0.18, -0.06) |  | -0.11 (-0.14, -0.09) |  | -0.20 (-0.23, -0.17) |  | -0.02 (-0.04, -0.00) |  |
| **Yes** | -0.24 (-0.34, -0.14) |  | -0.07 (-0.10, -0.04) |  | -0.11 (-0.13, -0.09) |  | -0.22 (-0.27, -0.17) |  | -0.04 (-0.06, -0.03) |  |
| **Hypertension** |  | 0.723 |  | 0.688 |  | 0.157 |  | 0.349 |  | 0.973 |
| **No** | -0.20 (-0.29, -0.11) |  | -0.08 (-0.12, -0.04) |  | -0.13 (-0.16, -0.09) |  | -0.22 (-0.27, -0.18) |  | -0.03 (-0.05, -0.02) |  |
| **Yes** | -0.19 (-0.30, -0.07) |  | -0.08 (-0.12, -0.05) |  | -0.10 (-0.12, -0.08) |  | -0.19 (-0.22, -0.15) |  | -0.03 (-0.05, -0.01) |  |
| **Stroke** |  | 0.963 |  | 0.995 |  | <0.001 |  | 0.124 |  | 0.099 |
| **No** | -0.18 (-0.26, -0.10) |  | -0.08 (-0.11, -0.05) |  | -0.10 (-0.12, -0.08) |  | -0.21 (-0.23, -0.18) |  | -0.03 (-0.04, -0.02) |  |
| **Yes** | -0.23 (-0.52, 0.06) |  | -0.09 (-0.19, 0.01) |  | -0.21 (-0.27, -0.14) |  | -0.11 (-0.26, 0.05) |  | -0.09 (-0.17, -0.01) |  |
| **Cancer** |  | 0.610 |  | 0.207 |  | 0.928 |  | 0.263 |  | 0.345 |
| **No** | -0.19 (-0.26, -0.11) |  | -0.07 (-0.10, -0.05) |  | -0.11 (-0.13, -0.09) |  | -0.20 (-0.23, -0.17) |  | -0.03 (-0.04, -0.02) |  |
| **Yes** | -0.09 (-0.39, 0.20) |  | -0.11 (-0.19, -0.04) |  | -0.10 (-0.14, -0.06) |  | -0.14 (-0.25, -0.02) |  | -0.06 (-0.10, -0.01) |  |
| **Depressive symptom** |  | 0.440 |  | 0.003 |  | 0.480 |  | 0.075 |  | 0.011 |
| **No** | -0.17 (-0.25, -0.09) |  | -0.06 (-0.09, -0.03) |  | -0.11 (-0.13, -0.10) |  | -0.19 (-0.23, -0.16) |  | -0.02 (-0.04, -0.01) |  |
| **Yes** | -0.26 (-0.45, -0.06) |  | -0.14 (-0.20, -0.09) |  | -0.10 (-0.14, -0.05) |  | -0.23 (-0.28, -0.18) |  | -0.07 (-0.10, -0.04) |  |

Notes: CHARLS: China Health and Retirement Longitudinal Study; ELSA: English Longitudinal Study of Ageing; HRS: Health and Retirement Study; MHAS: Mexican Health and Aging Study; SHARE: Survey of Health, Ageing and Retirement in Europe.

In addition to the stratification variables themselves, age, gender, education, labour force status, household wealth, married or partnered, co-residence with children, smoking, drinking, hypertension, stroke, cancer and depressive symptoms.

#The model failed because of the small sample size.

*For MHAS, the question on retirement was unavailable, so labour force status was recoded into currently working and currently not working.

| **Supplementary Table S10 Association between digital exclusion and memory by age, gender, education, labour force status, household wealth, married or partnered, co-residence with children, smoking, drinking, hypertension, stroke, cancer and depressive symptoms** | | | | | | | | | | |
| --- | --- | --- | --- | --- | --- | --- | --- | --- | --- | --- |
| **Subgroups** | **CHARLS** | | **ELSA** | | **HRS** | | **MHAS** | | **SHARE** | |
|  | **OR (95% CI)** | ***P* for interaction** | **OR (95% CI)** | ***P* for interaction** | **OR (95% CI)** | ***P* for interaction** | **OR (95% CI)** | ***P* for interaction** | **OR (95% CI)** | ***P* for interaction** |
| **Age** |  | 0.220 |  | 0.690 |  | <0.001 |  | 0.050 |  | 0.008 |
| **60-79** | -1.61 (-1.95, -1.27) |  | -1.10 (-1.27, -0.93) |  | -1.04 (-1.14, -0.94) |  | -0.77 (-0.88, -0.67) |  | -1.38 (-1.48, -1.29) |  |
| **≥80** | -3.37 (-5.24, -1.50) |  | -1.09 (-1.41, -0.78) |  | -1.36 (-1.53, -1.20) |  | -0.58 (-0.84, -0.33) |  | -1.84 (-2.16, -1.52) |  |
| **Gender** |  | 0.258 |  | 0.165 |  | 0.977 |  | 0.775 |  | 0.008 |
| **Male** | -1.26 (-1.68, -0.84) |  | -0.89 (-1.10, -0.68) |  | -0.97 (-1.09, -0.85) |  | -0.75 (-0.89, -0.60) |  | -1.06 (-1.20, -0.93) |  |
| **Female** | -1.57 (-2.08, -1.07) |  | -0.85 (-1.06, -0.64) |  | -0.94 (-1.06, -0.83) |  | -0.71 (-0.82, -0.59) |  | -1.32 (-1.45, -1.19) |  |
| **Education** |  | <0.001 |  | 0.049 |  | <0.001 |  | 0.240 |  | <0.001 |
| **Less than upper secondary** | -1.93 (-2.32, -1.53) |  | -0.90 (-1.13, -0.67) |  | -0.75 (-0.99, -0.52) |  | -0.75 (-0.84, -0.65) |  | -1.38 (-1.52, -1.24) |  |
| **Upper secondary and vocational training** | -0.52 (-1.09, 0.05) |  | -0.85 (-1.06, -0.65) |  | -0.87 (-1.00, -0.74) |  | -0.23 (-0.80, 0.34) |  | -1.16 (-1.30, -1.02) |  |
| **Tertiary** | -0.18 (-1.48, 1.11) |  | -1.07 (-1.65, -0.50) |  | -1.07 (-1.19, -0.94) |  | -0.61 (-0.98, -0.25) |  | -0.87 (-1.10, -0.64) |  |
| **Labour force status** |  | 0.154 |  | 0.201 |  | <0.001 |  | 0.692 |  | 0.248 |
| **Currently not working** | -1.36 (-1.74, -0.98) |  | -0.92 (-1.08, -0.76) |  | -1.02 (-1.12, -0.93) |  | -0.74 (-0.85, -0.64) |  | -1.20 (-1.30, -1.10) |  |
| **Currently working without retirement** | -1.65 (-2.46, -0.83) |  | -0.59 (-1.03, -0.15) |  | -0.71 (-1.01, -0.41) |  | -0.76 (-0.93, -0.59) |  | -1.27 (-1.57, -0.98) |  |
| **Currently working after retirement** | -0.69 (-1.35, -0.02) |  | -0.62 (-1.58, 0.34) |  | -0.78 (-1.01, -0.55) |  | —* |  | -0.85 (-1.26, -0.44) |  |
| **Household wealth** |  | 0.620 |  | 0.263 |  | 0.004 |  | 0.129 |  | 0.024 |
| **Low tertile** | -1.54 (-2.27, -0.81) |  | -1.03 (-1.25, -0.81) |  | -1.18 (-1.33, -1.04) |  | -0.84 (-1.00, -0.67) |  | -1.02 (-1.19, -0.84) |  |
| **Medium tertile** | -1.54 (-2.32, -0.76) |  | -0.90 (-1.16, -0.65) |  | -0.95 (-1.08, -0.81) |  | -0.71 (-0.87, -0.55) |  | -1.22 (-1.38, -1.07) |  |
| **High tertile** | -1.28 (-1.66, -0.90) |  | -0.75 (-1.06, -0.44) |  | -0.93 (-1.07, -0.80) |  | -0.65 (-0.79, -0.50) |  | -1.34 (-1.50, -1.18) |  |
| **Married or partnered** |  | 0.971 |  | 0.324 |  | 0.174 |  | 0.736 |  | 0.020 |
| **No** | -1.50 (-2.26, -0.73) |  | -0.98 (-1.23, -0.73) |  | -1.04 (-1.18, -0.90) |  | -0.68 (-0.83, -0.53) |  | -1.43 (-1.66, -1.21) |  |
| **Yes** | -1.42 (-1.78, -1.06) |  | -0.81 (-0.99, -0.62) |  | —# |  | -0.76 (-0.87, -0.64) |  | -1.14 (-1.25, -1.04) |  |
| **Co-residence with children** |  | 0.733 |  | 0.892 |  | 0.523 |  | 0.028 |  | 0.686 |
| **No** | -1.37 (-1.78, -0.97) |  | -0.87 (-1.02, -0.72) |  | —# |  | -0.87 (-1.05, -0.70) |  | -1.20 (-1.30, -1.09) |  |
| **Yes** | -1.52 (-2.04, -1.00) |  | -0.73 (-2.64, 1.17) |  | -0.95 (-1.05, -0.86) |  | -0.68 (-0.79, -0.57) |  | -1.14 (-1.38, -0.91) |  |
| **Smoking** |  | 0.263 |  | 0.217 |  | 0.944 |  | 0.141 |  | 0.287 |
| **No** | -1.49 (-1.86, -1.11) |  | -0.90 (-1.05, -0.74) |  | -0.96 (-1.05, -0.88) |  | -0.74 (-0.84, -0.65) |  | -1.21 (-1.32, -1.11) |  |
| **Yes** | -1.17 (-1.70, -0.64) |  | -0.68 (-1.16, -0.20) |  | -0.93 (-1.21, -0.64) |  | -0.60 (-0.89, -0.32) |  | -1.07 (-1.29, -0.84) |  |
| **Drinking** |  | 0.578 |  | 0.271 |  | 0.910 |  | 0.269 |  | 0.026 |
| **No** | -1.33 (-1.79, -0.86) |  | -1.08 (-1.42, -0.73) |  | -0.95 (-1.08, -0.83) |  | -0.72 (-0.83, -0.62) |  | -1.08 (-1.21, -0.94) |  |
| **Yes** | -1.52 (-1.99, -1.05) |  | -0.85 (-1.01, -0.68) |  | -1.00 (-1.11, -0.89) |  | -0.91 (-1.10, -0.72) |  | -1.29 (-1.41, -1.16) |  |
| **Hypertension** |  | 0.612 |  | 0.875 |  | <0.001 |  | 0.012 |  | 0.493 |
| **No** | -1.52 (-1.99, -1.05) |  | -0.85 (-1.07, -0.62) |  | -1.12 (-1.27, -0.98) |  | -0.92 (-1.08, -0.77) |  | -1.22 (-1.35, -1.09) |  |
| **Yes** | -1.28 (-1.73, -0.83) |  | -0.90 (-1.10, -0.70) |  | -0.90 (-1.00, -0.79) |  | -0.65 (-0.77, -0.54) |  | -1.15 (-1.29, -1.02) |  |
| **Stroke** |  | 0.190 |  | 0.125 |  | 0.124 |  | 0.209 |  | 0.009 |
| **No** | -1.37 (-1.71, -1.03) |  | -0.84 (-0.99, -0.69) |  | -0.94 (-1.03, -0.85) |  | -0.74 (-0.83, -0.65) |  | -1.16 (-1.26, -1.07) |  |
| **Yes** | -2.09 (-3.18, -1.00) |  | -1.34 (-1.91, -0.77) |  | -1.18 (-1.44, -0.91) |  | -0.54 (-0.98, -0.11) |  | -1.74 (-2.18, -1.30) |  |
| **Cancer** |  | 0.543 |  | 0.167 |  | 0.013 |  | 0.592 |  | 0.341 |
| **No** | -1.42 (-1.74, -1.10) |  | -0.85 (-1.01, -0.68) |  | -1.00 (-1.10, -0.91) |  | -0.73 (-0.82, -0.63) |  | -1.18 (-1.27, -1.08) |  |
| **Yes** | -0.90 (-2.66, 0.86) |  | -1.05 (-1.41, -0.69) |  | -0.79 (-0.98, -0.61) |  | -0.79 (-1.19, -0.39) |  | -1.33 (-1.62, -1.04) |  |
| **Depressive symptom** |  | 0.698 |  | 0.124 |  | 0.258 |  | 0.120 |  | 0.002 |
| **No** | -1.43 (-1.79, -1.07) |  | -0.84 (-1.01, -0.67) |  | -0.99 (-1.08, -0.90) |  | -0.79 (-0.90, -0.68) |  | -1.12 (-1.22, -1.01) |  |
| **Yes** | -1.18 (-1.94, -0.42) |  | -1.24 (-1.55, -0.93) |  | -0.96 (-1.15, -0.78) |  | -0.69 (-0.85, -0.53) |  | -1.47 (-1.67, -1.27) |  |

Notes: CHARLS: China Health and Retirement Longitudinal Study; ELSA: English Longitudinal Study of Ageing; HRS: Health and Retirement Study; MHAS: Mexican Health and Aging Study; SHARE: Survey of Health, Ageing and Retirement in Europe.

In addition to the stratification variables themselves, age, gender, education, labour force status, household wealth, married or partnered, co-residence with children, smoking, drinking, hypertension, stroke, cancer and depressive symptoms.

#The model failed because of the small sample size.

*For MHAS, the question on retirement was unavailable, so labour force status was recoded into currently working and currently not working.

| **Supplementary Table S11 Association between digital exclusion and** **executive by age, gender, education, labour force status, household wealth, married or partnered, co-residence with children, smoking, drinking, hypertension, stroke, cancer and depressive symptoms** | | | | | | | | | | |
| --- | --- | --- | --- | --- | --- | --- | --- | --- | --- | --- |
| **Subgroups** | **CHARLS** | | **ELSA** | | **HRS** | | **MHAS** | | **SHARE** | |
|  | **OR (95% CI)** | ***P* for interaction** | **OR (95% CI)** | ***P* for interaction** | **OR (95% CI)** | ***P* for interaction** | **OR (95% CI)** | ***P* for interaction** | **OR (95% CI)** | ***P* for interaction** |
| **Age** |  | 0.926 |  | 0.984 |  | 0.028 |  | 0.749 |  | 0.097 |
| **60-79** | -0.37 (-0.47, -0.28) |  | -0.21 (-0.27, -0.15) |  | -0.39 (-0.44, -0.35) |  | -0.38 (-0.43, -0.32) |  | -0.33 (-0.36, -0.29) |  |
| **≥80** | -0.18 (-0.96, 0.60) |  | -0.23 (-0.33, -0.13) |  | -0.38 (-0.46, -0.30) |  | -0.40 (-0.54, -0.26) |  | -0.44 (-0.56, -0.33) |  |
| **Gender** |  | 0.065 |  | 0.138 |  | 0.008 |  | 0.645 |  | 0.087 |
| **Male** | -0.22 (-0.33, -0.10) |  | -0.17 (-0.24, -0.10) |  | -0.31 (-0.37, -0.26) |  | -0.38 (-0.46, -0.30) |  | -0.26 (-0.30, -0.21) |  |
| **Female** | -0.46 (-0.65, -0.27) |  | -0.23 (-0.30, -0.15) |  | -0.39 (-0.44, -0.33) |  | -0.36 (-0.43, -0.29) |  | -0.32 (-0.37, -0.27) |  |
| **Education** |  | 0.346 |  | 0.013 |  | <0.001 |  | 0.013 |  | <0.001 |
| **Less than upper secondary** | -0.37 (-0.52, -0.22) |  | -0.26 (-0.35, -0.17) |  | -0.51 (-0.62, -0.39) |  | -0.39 (-0.45, -0.33) |  | -0.41 (-0.47, -0.35) |  |
| **Upper secondary and vocational training** | -0.21 (-0.37, -0.06) |  | -0.14 (-0.21, -0.07) |  | -0.30 (-0.37, -0.23) |  | -0.19 (-0.48, 0.10) |  | -0.22 (-0.27, -0.17) |  |
| **Tertiary** | -0.45 (-0.82, -0.08) |  | -0.24 (-0.43, -0.05) |  | -0.39 (-0.45, -0.33) |  | -0.16 (-0.35, 0.02) |  | -0.24 (-0.31, -0.17) |  |
| **Labour force status** |  | 0.039 |  | 0.303 |  | 0.011 |  | 0.481 |  | 0.974 |
| **Currently not working** | -0.39 (-0.51, -0.27) |  | -0.20 (-0.26, -0.15) |  | -0.38 (-0.42, -0.34) |  | -0.39 (-0.46, -0.33) |  | -0.29 (-0.33, -0.25) |  |
| **Currently working without retirement** | 0.09 (-0.16, 0.34) |  | -0.12 (-0.29, 0.06) |  | -0.29 (-0.42, -0.16) |  | -0.36 (-0.46, -0.27) |  | -0.30 (-0.40, -0.19) |  |
| **Currently working after retirement** | -0.35 (-0.59, -0.11) |  | -0.16 (-0.53, 0.22) |  | -0.41 (-0.52, -0.29) |  | —* |  | -0.31 (-0.46, -0.16) |  |
| **Household wealth** |  | 0.973 |  | 0.989 |  | <0.001 |  | 0.212 |  | 0.016 |
| **Low tertile** | -0.28 (-0.56, 0.00) |  | -0.21 (-0.29, -0.13) |  | -0.45 (-0.52, -0.38) |  | -0.40 (-0.49, -0.30) |  | -0.24 (-0.30, -0.18) |  |
| **Medium tertile** | -0.36 (-0.59, -0.12) |  | -0.20 (-0.29, -0.12) |  | -0.35 (-0.42, -0.29) |  | -0.31 (-0.40, -0.21) |  | -0.28 (-0.34, -0.23) |  |
| **High tertile** | -0.33 (-0.45, -0.21) |  | -0.19 (-0.28, -0.09) |  | -0.34 (-0.40, -0.28) |  | -0.40 (-0.48, -0.31) |  | -0.38 (-0.43, -0.32) |  |
| **Married or partnered** |  | 0.950 |  | 0.387 |  | 0.002 |  | 0.260 |  | 0.026 |
| **No** | -0.31 (-0.66, 0.04) |  | -0.23 (-0.32, -0.14) |  | -0.40 (-0.47, -0.33) |  | -0.35 (-0.44, -0.26) |  | -0.39 (-0.47, -0.31) |  |
| **Yes** | -0.32 (-0.42, -0.22) |  | -0.19 (-0.25, -0.12) |  | —# |  | -0.39 (-0.46, -0.33) |  | -0.28 (-0.31, -0.24) |  |
| **Co-residence with children** |  | 0.484 |  | 0.042 |  | 0.027 |  | 0.060 |  | 0.025 |
| **No** | -0.29 (-0.42, -0.16) |  | -0.20 (-0.25, -0.15) |  | —# |  | -0.43 (-0.53, -0.33) |  | -0.31 (-0.35, -0.28) |  |
| **Yes** | -0.40 (-0.57, -0.22) |  | 0.49 (-0.11, 1.08) |  | -0.37 (-0.41, -0.32) |  | -0.35 (-0.41, -0.29) |  | -0.19 (-0.28, -0.10) |  |
| **Smoking** |  | 0.379 |  | 0.003 |  | 0.443 |  | 0.981 |  | 0.474 |
| **No** | -0.35 (-0.48, -0.22) |  | -0.22 (-0.28, -0.16) |  | -0.36 (-0.40, -0.32) |  | -0.37 (-0.43, -0.31) |  | -0.30 (-0.33, -0.26) |  |
| **Yes** | -0.20 (-0.39, -0.02) |  | -0.01 (-0.16, 0.13) |  | -0.35 (-0.49, -0.20) |  | -0.38 (-0.54, -0.22) |  | -0.25 (-0.33, -0.18) |  |
| **Drinking** |  | 0.619 |  | 0.387 |  | 0.149 |  | 0.108 |  | 0.071 |
| **No** | -0.35 (-0.50, -0.19) |  | -0.25 (-0.37, -0.12) |  | -0.37 (-0.43, -0.31) |  | -0.36 (-0.42, -0.30) |  | -0.26 (-0.31, -0.20) |  |
| **Yes** | -0.29 (-0.44, -0.15) |  | -0.19 (-0.25, -0.14) |  | -0.37 (-0.42, -0.32) |  | -0.49 (-0.60, -0.38) |  | -0.33 (-0.37, -0.28) |  |
| **Hypertension** |  | 0.945 |  | 0.915 |  | 0.212 |  | 0.025 |  | 0.776 |
| **No** | -0.31 (-0.43, -0.19) |  | -0.18 (-0.25, -0.11) |  | -0.34 (-0.41, -0.27) |  | -0.44 (-0.53, -0.35) |  | -0.29 (-0.33, -0.24) |  |
| **Yes** | -0.34 (-0.51, -0.16) |  | -0.22 (-0.30, -0.15) |  | -0.38 (-0.42, -0.33) |  | -0.34 (-0.40, -0.27) |  | -0.30 (-0.35, -0.25) |  |
| **Stroke** |  | 0.514 |  | 0.389 |  | 0.303 |  | 0.534 |  | 0.141 |
| **No** | -0.30 (-0.40, -0.20) |  | -0.20 (-0.25, -0.14) |  | -0.35 (-0.39, -0.31) |  | -0.38 (-0.43, -0.32) |  | -0.29 (-0.32, -0.25) |  |
| **Yes** | -0.54 (-0.97, -0.10) |  | -0.13 (-0.33, 0.07) |  | -0.46 (-0.59, -0.34) |  | -0.34 (-0.58, -0.09) |  | -0.42 (-0.58, -0.25) |  |
| **Cancer** |  | 0.986 |  | 0.088 |  | 0.06 |  | 0.701 |  | 0.583 |
| **No** | -0.31 (-0.41, -0.22) |  | -0.22 (-0.27, -0.16) |  | -0.37 (-0.42, -0.33) |  | -0.38 (-0.43, -0.32) |  | -0.29 (-0.33, -0.25) |  |
| **Yes** | -0.32 (-0.99, 0.34) |  | -0.13 (-0.25, -0.01) |  | -0.31 (-0.40, -0.23) |  | -0.28 (-0.53, -0.04) |  | -0.33 (-0.44, -0.23) |  |
| **Depressive symptom** |  | 0.427 |  | 0.651 |  | 0.988 |  | 0.440 |  | <0.001 |
| **No** | -0.35 (-0.44, -0.26) |  | -0.19 (-0.25, -0.13) |  | -0.39 (-0.43, -0.34) |  | -0.36 (-0.43, -0.30) |  | -0.24 (-0.28, -0.21) |  |
| **Yes** | -0.16 (-0.48, 0.16) |  | -0.25 (-0.37, -0.13) |  | -0.42 (-0.51, -0.33) |  | -0.44 (-0.53, -0.34) |  | -0.49 (-0.58, -0.41) |  |

Notes: CHARLS: China Health and Retirement Longitudinal Study; ELSA: English Longitudinal Study of Ageing; HRS: Health and Retirement Study; MHAS: Mexican Health and Aging Study; SHARE: Survey of Health, Ageing and Retirement in Europe.

In addition to the stratification variables themselves, age, gender, education, labour force status, household wealth, married or partnered, co-residence with children, smoking, drinking, hypertension, stroke, cancer and depressive symptoms.

#The model failed because of the small sample size.

*For MHAS, the question on retirement was unavailable, so labour force status was recoded into currently working and currently not working.

| **Supplementary Table S12 Association between digital exclusion and total cognitive scores by age, gender, education, labour force status, household wealth, married or partnered, co-residence with children, smoking, drinking, hypertension, stroke, cancer and depressive symptoms** | | | | | | | | | | |
| --- | --- | --- | --- | --- | --- | --- | --- | --- | --- | --- |
| **Subgroups** | **CHARLS** | | **ELSA** | | **HRS** | | **MHAS** | | **SHARE** | |
|  | **OR (95% CI)** | ***P* for interaction** | **OR (95% CI)** | ***P* for interaction** | **OR (95% CI)** | ***P* for interaction** | **OR (95% CI)** | ***P* for interaction** | **OR (95% CI)** | ***P* for interaction** |
| **Age** |  | 0.247 |  | 0.406 |  | <0.001 |  | 0.179 |  | <0.001 |
| **60-79** | -2.18 (-2.59, -1.78) |  | -1.34 (-1.54, -1.15) |  | -1.46 (-1.59, -1.34) |  | -1.24 (-1.38, -1.11) |  | -1.75 (-1.86, -1.64) |  |
| **≥80** | -4.31 (-6.55, -2.07) |  | -1.48 (-1.84, -1.12) |  | -1.96 (-2.18, -1.74) |  | -1.15 (-1.51, -0.78) |  | -2.47 (-2.86, -2.08) |  |
| **Gender** |  | 0.103 |  | 0.312 |  | 0.565 |  | 0.812 |  | 0.005 |
| **Male** | -1.64 (-2.14, -1.14) |  | -1.14 (-1.39, -0.90) |  | -1.35 (-1.50, -1.19) |  | -1.22 (-1.41, -1.03) |  | -1.35 (-1.51, -1.20) |  |
| **Female** | -2.20 (-2.84, -1.57) |  | -1.11 (-1.35, -0.88) |  | -1.34 (-1.49, -1.20) |  | -1.18 (-1.34, -1.02) |  | -1.68 (-1.83, -1.53) |  |
| **Education** |  | <0.001 |  | 0.019 |  | <0.001 |  | 0.015 |  | <0.001 |
| **Less than upper secondary** | -2.58 (-3.07, -2.08) |  | -1.22 (-1.49, -0.95) |  | -1.14 (-1.45, -0.83) |  | -1.24 (-1.37, -1.11) |  | -1.82 (-1.99, -1.64) |  |
| **Upper secondary and vocational training** | -0.81 (-1.49, -0.14) |  | -1.05 (-1.28, -0.81) |  | -1.23 (-1.39, -1.06) |  | -0.58 (-1.25, 0.10) |  | -1.42 (-1.58, -1.26) |  |
| **Tertiary** | -0.40 (-1.92, 1.12) |  | -1.43 (-2.09, -0.77) |  | -1.54 (-1.70, -1.39) |  | -0.89 (-1.33, -0.45) |  | -1.16 (-1.42, -0.90) |  |
| **Labour force status** |  | 0.276 |  | 0.114 |  | <0.001 |  | 0.385 |  | 0.449 |
| **Currently not working** | -1.86 (-2.31, -1.40) |  | -1.18 (-1.36, -1.00) |  | -1.44 (-1.56, -1.32) |  | -1.25 (-1.39, -1.10) |  | -1.52 (-1.64, -1.40) |  |
| **Currently working without retirement** | -1.89 (-2.92, -0.86) |  | -0.76 (-1.27, -0.26) |  | -1.01 (-1.38, -0.65) |  | -1.25 (-1.48, -1.02) |  | -1.57 (-1.92, -1.23) |  |
| **Currently working after retirement** | -1.16 (-1.87, -0.44) |  | -0.80 (-1.91, 0.31) |  | -1.26 (-1.55, -0.97) |  | —* |  | -1.18 (-1.65, -0.70) |  |
| **Household wealth** |  | 0.458 |  | 0.329 |  | <0.001 |  | 0.068 |  | 0.001 |
| **Low tertile** | -2.02 (-2.98, -1.06) |  | -1.32 (-1.58, -1.07) |  | -1.62 (-1.81, -1.42) |  | -1.37 (-1.60, -1.15) |  | -1.25 (-1.45, -1.05) |  |
| **Medium tertile** | -2.23 (-3.10, -1.37) |  | -1.15 (-1.45, -0.86) |  | -1.35 (-1.52, -1.18) |  | -1.17 (-1.39, -0.95) |  | -1.54 (-1.72, -1.36) |  |
| **High tertile** | -1.73 (-2.18, -1.27) |  | -0.99 (-1.35, -0.64) |  | -1.36 (-1.53, -1.19) |  | -1.07 (-1.27, -0.88) |  | -1.79 (-1.98, -1.60) |  |
| **Married or partnered** |  | 0.905 |  | 0.304 |  | 0.049 |  | 0.548 |  | 0.005 |
| **No** | -1.89 (-2.86, -0.92) |  | -1.28 (-1.56, -0.99) |  | -1.48 (-1.66, -1.30) |  | -1.12 (-1.33, -0.91) |  | -1.87 (-2.14, -1.61) |  |
| **Yes** | -1.93 (-2.35, -1.50) |  | -1.07 (-1.28, -0.85) |  | —# |  | -1.27 (-1.42, -1.12) |  | -1.45 (-1.57, -1.33) |  |
| **Co-residence with children** |  | 0.464 |  | 0.568 |  | 0.216 |  | 0.011 |  | 0.200 |
| **No** | -1.80 (-2.30, -1.31) |  | -1.14 (-1.31, -0.96) |  | —# |  | -1.41 (-1.64, -1.17) |  | -1.55 (-1.67, -1.43) |  |
| **Yes** | -2.19 (-2.83, -1.55) |  | -0.32 (-2.38, 1.73) |  | -1.35 (-1.48, -1.23) |  | -1.13 (-1.27, -0.99) |  | -1.34 (-1.61, -1.06) |  |
| **Smoking** |  | 0.254 |  | 0.029 |  | 0.421 |  | 0.224 |  | 0.206 |
| **No** | -2.01 (-2.47, -1.54) |  | -1.18 (-1.36, -1.00) |  | -1.37 (-1.48, -1.26) |  | -1.22 (-1.35, -1.09) |  | -1.55 (-1.67, -1.43) |  |
| **Yes** | -1.56 (-2.20, -0.92) |  | -0.74 (-1.28, -0.20) |  | -1.26 (-1.62, -0.89) |  | -1.07 (-1.46, -0.69) |  | -1.34 (-1.59, -1.08) |  |
| **Drinking** |  | 0.673 |  | 0.151 |  | 0.507 |  | 0.132 |  | 0.009 |
| **No** | -1.80 (-2.40, -1.21) |  | -1.44 (-1.83, -1.04) |  | -1.35 (-1.51, -1.20) |  | -1.19 (-1.33, -1.05) |  | -1.36 (-1.52, -1.19) |  |
| **Yes** | -2.03 (-2.59, -1.48) |  | -1.10 (-1.29, -0.91) |  | -1.44 (-1.58, -1.29) |  | -1.58 (-1.84, -1.33) |  | -1.66 (-1.81, -1.51) |  |
| **Hypertension** |  | 0.659 |  | 0.959 |  | 0.015 |  | 0.003 |  | 0.642 |
| **No** | -2.02 (-2.56, -1.47) |  | -1.08 (-1.34, -0.83) |  | -1.50 (-1.69, -1.32) |  | -1.49 (-1.70, -1.29) |  | -1.54 (-1.69, -1.39) |  |
| **Yes** | -1.79 (-2.34, -1.24) |  | -1.19 (-1.42, -0.96) |  | -1.31 (-1.44, -1.18) |  | -1.08 (-1.24, -0.93) |  | -1.49 (-1.64, -1.33) |  |
| **Stroke** |  | 0.221 |  | 0.262 |  | 0.023 |  | 0.125 |  | 0.004 |
| **No** | -1.83 (-2.24, -1.42) |  | -1.10 (-1.28, -0.92) |  | -1.32 (-1.43, -1.21) |  | -1.22 (-1.35, -1.10) |  | -1.48 (-1.59, -1.37) |  |
| **Yes** | -2.84 (-4.12, -1.56) |  | -1.55 (-2.19, -0.91) |  | -1.76 (-2.11, -1.41) |  | -0.93 (-1.52, -0.34) |  | -2.25 (-2.79, -1.71) |  |
| **Cancer** |  | 0.559 |  | 0.353 |  | 0.008 |  | 0.986 |  | 0.276 |
| **No** | -1.91 (-2.29, -1.52) |  | -1.12 (-1.30, -0.93) |  | -1.41 (-1.53, -1.29) |  | -1.21 (-1.34, -1.09) |  | -1.50 (-1.61, -1.38) |  |
| **Yes** | -1.43 (-3.83, 0.97) |  | -1.27 (-1.69, -0.86) |  | -1.14 (-1.38, -0.90) |  | -1.06 (-1.62, -0.51) |  | -1.71 (-2.05, -1.37) |  |
| **Depressive symptom** |  | 0.699 |  | 0.055 |  | 0.319 |  | 0.671 |  | <0.001 |
| **No** | -1.94 (-2.37, -1.51) |  | -1.06 (-1.26, -0.87) |  | -1.43 (-1.55, -1.32) |  | -1.26 (-1.41, -1.12) |  | -1.39 (-1.51, -1.27) |  |
| **Yes** | -1.55 (-2.47, -0.62) |  | -1.65 (-2.00, -1.29) |  | -1.42 (-1.66, -1.18) |  | -1.31 (-1.53, -1.08) |  | -2.04 (-2.28, -1.79) |  |

Notes: CHARLS: China Health and Retirement Longitudinal Study; ELSA: English Longitudinal Study of Ageing; HRS: Health and Retirement Study; MHAS: Mexican Health and Aging Study; SHARE: Survey of Health, Ageing and Retirement in Europe.

In addition to the stratification variables themselves, age, gender, education, labour force status, household wealth, married or partnered, co-residence with children, smoking, drinking, hypertension, stroke, cancer and depressive symptoms.

#The model failed because of the small sample size.

*For MHAS, the question on retirement was unavailable, so labour force status was recoded into currently working and currently not working.

| **Supplementary Table S13 Sensitivity analysis of the association between digital exclusion and cognitive impairment was performed by interpolating the missing covariates** | | | | | | |
| --- | --- | --- | --- | --- | --- | --- |
|  |  | **CHARLS** | **ELSA** | **HRS** | **MHAS** | **SHARE** |
|  |  | **OR (95% CI) P value** | **OR (95% CI) P value** | **OR (95% CI) P value** | **OR (95% CI) P value** | **OR (95% CI) P value** |
| **Cognitive impairment** | **Model 1** | 4.36 (3.51, 5.42) <0.001 | 2.56 (2.34, 2.80) <0.001 | 4.15 (3.86, 4.47) <0.001 | 2.46 (2.25, 2.70) <0.001 | 4.70 (4.44, 4.96) <0.001 |
|  | **Model 2** | 3.07 (2.32, 4.06) <0.001 | 2.03 (1.84, 2.24) <0.001 | 2.63 (2.41, 2.86) <0.001 | 2.00 (1.82, 2.20) <0.001 | 3.14 (2.95, 3.34) <0.001 |
|  | **Model 3** | 2.90 (2.16, 3.90) <0.001 | 1.91 (1.72, 2.11) <0.001 | 2.48 (2.27, 2.70) <0.001 | 2.02 (1.84, 2.22) <0.001 | 3.03 (2.85, 3.23) <0.001 |
| **Orientation scores** | **Model 1** | -0.47 (-0.54, -0.39) <0.001 | -0.16 (-0.18, -0.14) <0.001 | -0.26 (-0.27, -0.24) <0.001 | -0.29 (-0.31, -0.26) <0.001 | -0.13 (-0.13, -0.12) <0.001 |
|  | **Model 2** | -0.22 (-0.28, -0.16) <0.001 | -0.10 (-0.12, -0.08) <0.001 | -0.13 (-0.14, -0.11) <0.001 | -0.21 (-0.23, -0.18) <0.001 | -0.05 (-0.06, -0.05) <0.001 |
|  | **Model 3** | -0.19 (-0.24, -0.13) <0.001 | -0.09 (-0.11, -0.07) <0.001 | -0.11 (-0.13, -0.09) <0.001 | -0.21 (-0.24, -0.18) <0.001 | -0.05 (-0.06, -0.04) <0.001 |
| **Memory scores** | **Model 1** | -2.50 (-2.77, -2.23) <0.001 | -1.88 (-2.01, -1.75) <0.001 | -2.08 (-2.17, -2.00) <0.001 | -1.18 (-1.27, -1.09) <0.001 | -2.76 (-2.81, -2.71) <0.001 |
|  | **Model 2** | -1.56 (-1.81, -1.31) <0.001 | -0.87 (-0.99, -0.75) <0.001 | -1.04 (-1.12, -0.96) <0.001 | -0.77 (-0.86, -0.68) <0.001 | -1.42 (-1.48, -1.36) <0.001 |
|  | **Model 3** | -1.44 (-1.68, -1.20) <0.001 | -0.78 (-0.90, -0.67) <0.001 | -0.96 (-1.04, -0.88) <0.001 | -0.77 (-0.86, -0.69) <0.001 | -1.36 (-1.41, -1.30) <0.001 |
| **Executive scores** | **Model 1** | -0.75 (-0.83, -0.67) <0.001 | -0.35 (-0.39, -0.31) <0.001 | -0.76 (-0.80, -0.72) <0.001 | -0.58 (-0.63, -0.53) <0.001 | -0.69 (-0.71, -0.68) <0.001 |
|  | **Model 2** | -0.38 (-0.46, -0.29) <0.001 | -0.20 (-0.24, -0.16) <0.001 | -0.39 (-0.43, -0.35) <0.001 | -0.38 (-0.43, -0.33) <0.001 | -0.33 (-0.35, -0.31) <0.001 |
|  | **Model 3** | -0.34 (-0.42, -0.25) <0.001 | -0.18 (-0.22, -0.14) <0.001 | -0.36 (-0.40, -0.32) <0.001 | -0.38 (-0.43, -0.33) <0.001 | -0.32 (-0.34, -0.30) <0.001 |
| **Total cognitive scores** | **Model 1** | -3.67 (-4.02, -3.31) <0.001 | -2.34 (-2.49, -2.18) <0.001 | -2.96 (-3.08, -2.85) <0.001 | -1.87 (-2.00, -1.74) <0.001 | -3.58 (-3.64, -3.51) <0.001 |
|  | **Model 2** | -2.13 (-2.45, -1.81) <0.001 | -1.12 (-1.26, -0.98) <0.001 | -1.46 (-1.57, -1.36) <0.001 | -1.25 (-1.36, -1.13) <0.001 | -1.80 (-1.87, -1.73) <0.001 |
|  | **Model 3** | -1.95 (-2.26, -1.64) <0.001 | -1.02 (-1.15, -0.88) <0.001 | -1.36 (-1.46, -1.25) <0.001 | -1.26 (-1.38, -1.14) <0.001 | -1.72 (-1.79, -1.65) <0.001 |

Notes: CHARLS: China Health and Retirement Longitudinal Study; ELSA: English Longitudinal Study of Ageing; HRS: Health and Retirement Study; MHAS: Mexican Health and Aging Study; SHARE: Survey of Health, Ageing and Retirement in Europe.

Model 1: No variables are adjusted;

Model 2: Adjusted for the minimal sufficient adjustment set (MSAS) identified using a causal directed acyclic graph (DAG) including further adjusted for age, gender, education, labour force status, household wealth, married or partnered and co-residence with children;

Model 3: Further adjusted for smoking, drinking, hypertension, stroke, cancer, and depressive symptom based on Model 2.

| **Supplementary Table S14 Sensitivity analysis of the association between digital exclusion and cognitive impairment was performed by excluding participants with cognitive impairment at baseline** | | | | | | |
| --- | --- | --- | --- | --- | --- | --- |
|  |  | **CHARLS** | **ELSA** | **HRS** | **MHAS** | **SHARE** |
|  |  | **OR/β (95% CI) P value** | **OR/β (95% CI) P value** | **OR/β (95% CI) P value** | **OR/β (95% CI) P value** | **OR/β (95% CI) P value** |
| **Cognitive impairment** | **Model 1** | 4.22 (2.98, 5.97) <0.001 | 2.49 (2.20, 2.81) <0.001 | 4.68 (4.30, 5.10) <0.001 | 2.23 (1.95, 2.55) <0.001 | 5.56 (4.88, 6.34) <0.001 |
|  | **Model 2** | 2.66 (1.74, 4.07) <0.001 | 1.71 (1.49, 1.98) <0.001 | 2.67 (2.42, 2.94) <0.001 | 1.83 (1.59, 2.10) <0.001 | 3.11 (2.68, 3.60) <0.001 |
|  | **Model 3** | 2.54 (1.64, 3.91) <0.001 | 1.63 (1.42, 1.89) <0.001 | 2.48 (2.25, 2.74) <0.001 | 1.75 (1.52, 2.01) <0.001 | 2.96 (2.55, 3.44) <0.001 |
| **Orientation scores** | **Model 1** | -0.46 (-0.54, -0.39) <0.001 | -0.12 (-0.14, -0.09) <0.001 | -0.23 (-0.25, -0.22) <0.001 | -0.23 (-0.26, -0.21) <0.001 | -0.08 (-0.10, -0.07) <0.001 |
|  | **Model 2** | -0.19 (-0.27, -0.12) <0.001 | -0.06 (-0.09, -0.04) <0.001 | -0.11 (-0.13, -0.09) <0.001 | -0.16 (-0.19, -0.14) <0.001 | -0.02 (-0.04, -0.01) <0.001 |
|  | **Model 3** | -0.17 (-0.24, -0.10) <0.001 | -0.06 (-0.08, -0.03) <0.001 | -0.10 (-0.11, -0.08) <0.001 | -0.15 (-0.18, -0.13) <0.001 | -0.02 (-0.03, -0.01) 0.002 |
| **Memory scores** | **Model 1** | -2.54 (-2.88, -2.20) <0.001 | -2.11 (-2.26, -1.95) <0.001 | -2.03 (-2.11, -1.94) <0.001 | -1.02 (-1.11, -0.92) <0.001 | -2.39 (-2.48, -2.31) <0.001 |
|  | **Model 2** | -1.47 (-1.80, -1.14) <0.001 | -0.75 (-0.90, -0.60) <0.001 | -0.97 (-1.05, -0.89) <0.001 | -0.62 (-0.71, -0.53) <0.001 | -1.10 (-1.19, -1.01) <0.001 |
|  | **Model 3** | -1.36 (-1.69, -1.03) <0.001 | -0.69 (-0.84, -0.55) <0.001 | -0.89 (-0.97, -0.81) <0.001 | -0.57 (-0.66, -0.48) <0.001 | -1.03 (-1.12, -0.94) <0.001 |
| **Executive scores** | **Model 1** | -0.71 (-0.80, -0.63) <0.001 | -0.34 (-0.39, -0.29) <0.001 | -0.76 (-0.80, -0.72) <0.001 | -0.55 (-0.60, -0.50) <0.001 | -0.58 (-0.61, -0.55) <0.001 |
|  | **Model 2** | -0.33 (-0.43, -0.23) <0.001 | -0.17 (-0.23, -0.12) <0.001 | -0.37 (-0.40, -0.33) <0.001 | -0.35 (-0.40, -0.29) <0.001 | -0.23 (-0.27, -0.20) <0.001 |
|  | **Model 3** | -0.29 (-0.39, -0.20) <0.001 | -0.16 (-0.22, -0.11) <0.001 | -0.33 (-0.37, -0.29) <0.001 | -0.33 (-0.39, -0.28) <0.001 | -0.21 (-0.25, -0.18) <0.001 |
| **Total cognitive scores** | **Model 1** | -3.67 (-4.08, -3.27) <0.001 | -2.56 (-2.74, -2.37) <0.001 | -2.95 (-3.06, -2.84) <0.001 | -1.73 (-1.85, -1.60) <0.001 | -3.06 (-3.15, -2.96) <0.001 |
|  | **Model 2** | -1.97 (-2.38, -1.57) <0.001 | -0.97 (-1.14, -0.80) <0.001 | -1.38 (-1.49, -1.28) <0.001 | -1.09 (-1.21, -0.97) <0.001 | -1.36 (-1.46, -1.26) <0.001 |
|  | **Model 3** | -1.81 (-2.21, -1.42) <0.001 | -0.91 (-1.08, -0.74) <0.001 | -1.27 (-1.37, -1.17) <0.001 | -1.03 (-1.14, -0.91) <0.001 | -1.26 (-1.36, -1.16) <0.001 |

Notes: CHARLS: China Health and Retirement Longitudinal Study; ELSA: English Longitudinal Study of Ageing; HRS: Health and Retirement Study; MHAS: Mexican Health and Aging Study; SHARE: Survey of Health, Ageing and Retirement in Europe.

Model 1: No variables are adjusted;

Model 2: Adjusted for the minimal sufficient adjustment set (MSAS) identified using a causal directed acyclic graph (DAG) including further adjusted for age, gender, education, labour force status, household wealth, maORied or partnered and co-residence with children;

Model 3: Further adjusted for smoking, drinking, hypertension, stroke, cancer, and depressive symptom based on Model 2.


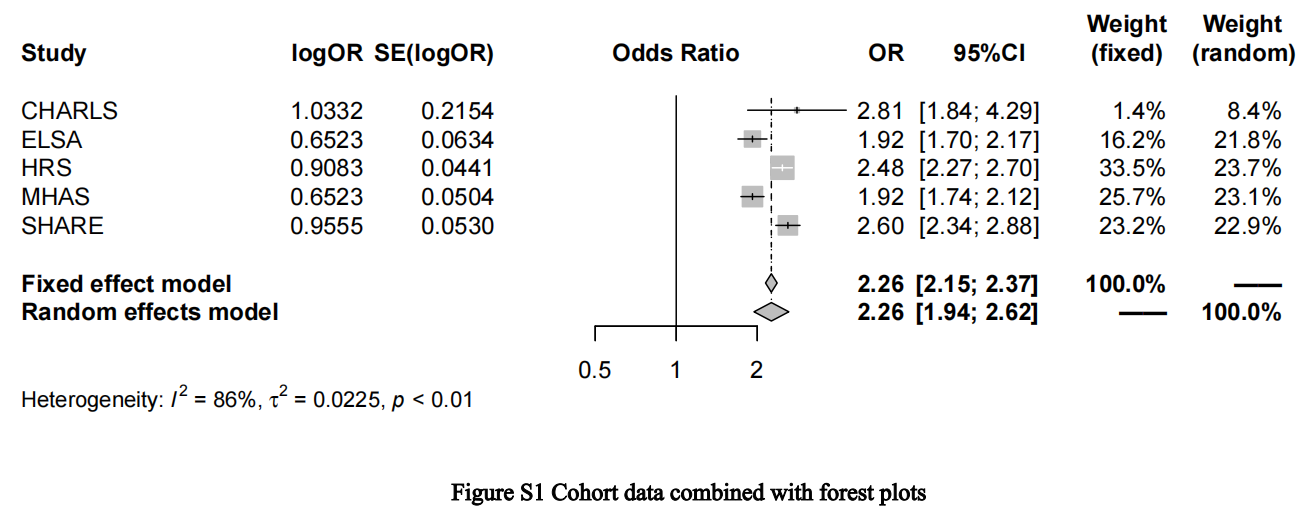


STROBE Statement—Checklist of items that should be included in reports of ***cross-sectional studies***

|  | Item No | Recommendation | Page  No |
| --- | --- | --- | --- |
| **Title and abstract** | 1 | (*a*) Indicate the study’s design with a commonly used term in the title or the abstract | 1 |
|  |  | (*b*) Provide in the abstract an informative and balanced summary of what was done and what was found | 1 |
| Introduction | | |  |
| Background/rationale | 2 | Explain the scientific background and rationale for the investigation being reported | 4-5 |
| Objectives | 3 | State specific objectives, including any prespecified hypotheses | 4-5 |
| Methods | | |  |
| Study design | 4 | Present key elements of study design early in the paper | 5 |
| Setting | 5 | Describe the setting, locations, and relevant dates, including periods of recruitment, exposure, follow-up, and data collection | 5 |
| Participants | 6 | (*a*) Give the eligibility criteria, and the sources and methods of selection of participants | 5 |
| Variables | 7 | Clearly define all outcomes, exposures, predictors, potential confounders, and effect modifiers. Give diagnostic criteria, if applicable | 6-8 |
| Data sources/ measurement | 8* | For each variable of interest, give sources of data and details of methods of assessment (measurement). Describe comparability of assessment methods if there is more than one group | 5-8 |
| Bias | 9 | Describe any efforts to address potential sources of bias | 8-9 |
| Study size | 10 | Explain how the study size was arrived at | 5 |
| Quantitative variables | 11 | Explain how quantitative variables were handled in the analyses. If applicable, describe which groupings were chosen and why | NA |
| Statistical methods | 12 | (*a*) Describe all statistical methods, including those used to control for confounding | 8-9 |
|  |  | (*b*) Describe any methods used to examine subgroups and interactions | 8-9 |
|  |  | (*c*) Explain how missing data were addressed | 9 |
|  |  | (*d*) If applicable, describe analytical methods taking account of sampling strategy | NA |
|  |  | (*e*) Describe any sensitivity analyses | 8-9 |
| Results | | |  |
| Participants | 13* | (a) Report numbers of individuals at each stage of study—eg numbers potentially eligible, examined for eligibility, confirmed eligible, included in the study, completing follow-up, and analysed | Figure 1 |
|  |  | (b) Give reasons for non-participation at each stage | 5 |
|  |  | (c) Consider use of a flow diagram | Figure 1 |
| Descriptive data | 14* | (a) Give characteristics of study participants (eg demographic, clinical, social) and information on exposures and potential confounders | Table 1 |
|  |  | (b) Indicate number of participants with missing data for each variable of interest | Table S4 |
| Outcome data | 15* | Report numbers of outcome events or summary measures | Table S3 |
| Main results | 16 | (*a*) Give unadjusted estimates and, if applicable, confounder-adjusted estimates and their precision (eg, 95% confidence interval). Make clear which confounders were adjusted for and why they were included | Table 2 |
|  |  | (*b*) Report category boundaries when continuous variables were categorized | NA |
|  |  | (*c*) If relevant, consider translating estimates of relative risk into absolute risk for a meaningful time period | NA |
| Other analyses | 17 | Report other analyses done—eg analyses of subgroups and interactions, and sensitivity analyses | Table S11-S16 |
| Discussion | | |  |
| Key results | 18 | Summarise key results with reference to study objectives | 12 |
| Limitations | 19 | Discuss limitations of the study, taking into account sources of potential bias or imprecision. Discuss both direction and magnitude of any potential bias | 15-16 |
| Interpretation | 20 | Give a cautious overall interpretation of results considering objectives, limitations, multiplicity of analyses, results from similar studies, and other relevant evidence | 12-16 |
| Generalisability | 21 | Discuss the generalisability (external validity) of the study results | 15-16 |
| Other information | | |  |
| Funding | 22 | Give the source of funding and the role of the funders for the present study and, if applicable, for the original study on which the present article is based | 17 |
